# Modulation of brain immune microenvironment and cellular dynamics in systemic inflammation

**DOI:** 10.7150/thno.107061

**Published:** 2025-04-09

**Authors:** Junhao Wang, Zhaoqian Zhong, Haihua Luo, Qizheng Han, Kan Wu, Aolin Jiang, Li Chen, Yanxia Gao, Yong Jiang

**Affiliations:** 1 Guangdong Provincial Key Laboratory of Proteomics, State Key Laboratory of Organ Failure Research, Department of Pathophysiology, School of Basic Medical Sciences, Southern Medical University, Guangzhou 510515, China.; 2 Henan Key Laboratory of Critical Care Medicine, Henan International Joint Laboratory of Infection and Immunity, Department of Critical Care Medicine and Department of Emergency Medicine, The First Affiliated Hospital of Zhengzhou University, Zhengzhou 450052, China.; 3 Institute of Infection and Immunity, Henan Academy of Innovations in Medical Science Zhengzhou 451163, China.; 4 Department of Respiratory and Critical Care Medicine, The Tenth Affiliated Hospital (Dongguan People's Hospital), Southern Medical University, Dongguan, 523059, China.; 5 Current address: Department of Medicine, Section of Epidemiology and Population Sciences, Dan L Duncan Comprehensive Cancer Center, Baylor College of Medicine, One Baylor Plaza, Houston, TX, USA.

**Keywords:** Sepsis, Neuroinflammatory microenvironment, Multi-omics analysis, Intercellular communication inferred

## Abstract

**Background:** Sepsis-associated encephalopathy (SAE) is a severe complication of sepsis, affecting approximately 70% of patients, leading to increased mortality and long-term cognitive impairments among survivors. However, there is a lack of comprehensive studies on the development of SAE, especially related to the cellular communication networks in the brain microenvironment.

**Methods:** We evaluated the impact of myeloid cells on the brain's immune microenvironment through glial cell alterations using bulk and single-cell transcriptomics data from human and mouse models and validated this with correlative experiments. We also developed the DeconvCellLink R package to study neuroinflammation-associated cellular interaction networks. A dynamic brain immune microenvironment map showing temporal alterations in brain cellular network during systemic inflammatory reactions was constructed using time-series data.

**Results:** While brain cellular alterations differed between human and animal models, a highly conserved set of sepsis-associated genes regulating immune microenvironment signalling was identified. The dynamic alterations in cellular interaction networks and cytokines revealed brain immune cells' temporal response to systemic inflammation. We also found that valproic acid could mitigate sepsis-induced neuroinflammation by regulating glial cell balance and modulating the neuroimmune microenvironment.

**Conclusion:** Through dynamic cellular communication networks, the study revealed that, immune dysregulation in the inflamed brain in SAE involves overactivation of innate immunity, with neutrophils playing a crucial role, providing a scientific framework for developing novel therapeutic strategies and offering new insights into the mechanisms underlying sepsis-induced brain dysfunction.

## Introduction

Sepsis is a life-threatening organ dysfunction resulting from a dysregulated host response to infection. Sepsis-associated encephalopathy (SAE) is a common and serious complication that affects approximately 70% of patients with severe sepsis and results in increased mortality rates and long-term cognitive impairment among survivors 1-4. Proinflammatory cytokines, mitochondrial dysfunction, and breakdown of the blood-brain barrier (BBB) are among the numerous factors that contribute to the development of SAEs; however, the precise mechanisms involved remain uncertain 5.

The immune microenvironment in SAE is regulated by various cell types, including brain endothelial cells, glial cells, neurons, and infiltrating immune cells. The BBB, compromised by neuroinflammation and ischemic injury caused by sepsis, allows peripheral immune cells such as inflammatory monocytes, macrophages, and neutrophils to infiltrate the central nervous system, activate resident microglia, and exacerbate neuroinflammation 5-7.

Although animal models have provided valuable insights into the mechanisms underlying SAE, studies on human SAE samples are limited. Animal studies on sepsis drugs have been inconsistent with human clinical trials, with none showing satisfactory results in clinical trials over the past 30 years 8, highlighting the need for further research on human and mouse data.

To identify sepsis-related genes that affect the immune microenvironment of the brain, we analyzed human sepsis brain samples and mouse endotoxemia brain models. We developed a method called DeconvCellLink, which integrates semi-supervised mouse data deconvolution, enrichment analysis, and Bayesian network inference to predict potential associations between deconvoluted cell types using mouse bulk RNA sequencing (RNA-seq) data. The R package DeconvCellLink is accessible at https://github.com/JH-42/DeconvCellLink.

We used DeconvCellLink to identify putative co-regulatory cell types and intercellular communication in the brains of mice with sepsis. We monitored the changes in brain cytokine activity, signaling pathway activity, and potential cell interactions over time during systemic inflammation. Additionally, we investigated the mechanisms of action of valproic acid (VPA) as a potential drug for treating brain neuroinflammation using our recently developed algorithm.

## Methods

### Data acquisition

Transcriptome RNA-seq count data from the grey matter of the parietal cortex of 24 patients (12 who died of sepsis and 12 from non-infectious critical diseases) 9 were collected from the ARCHS4 database 10. Gene expression datasets of whole brains from lipopolysaccharide (LPS)-challenged mice 11 and hippocampi from mice subjected to cecal ligation and puncture (CLP) 12 were downloaded from the NCBI Gene Expression Omnibus (GEO) datasets (GSE88959, GSE128925). Single-nucleus RNA sequencing (snRNA-seq) data of white matter 13 from patients with sepsis (MS242) and without sepsis (CO14) were downloaded from the GEO (GSE118257). Visium spatial gene expression data for the forebrains of C57BL/6 mice were downloaded from the 10× Genomics spatial gene expression datasets 14.

Data from the hippocampus and cortex of human sepsis samples were downloaded from the GEO (GSE237861) 15. Time-series whole-brain data from LPS-challenged mice were accessed from GSE224127 16, whereas spatial transcriptomic data for the whole body of LPS-challenged mice were obtained from the array-seq data in GSE248904 16.

Single-cell transcriptome data of the whole cortical and hippocampal regions of adult mice were acquired using the Allen Brain Map. The spatial transcriptomics of saline- and LPS-challenged mice (brain coronal sections) were downloaded from the GEO (GSE165098) 17. Additionally, single-cell transcriptome data for CD45^+^ cells isolated from the whole brain (parenchyma and border regions) of LPS-challenged mice were obtained from the GEO (GSE157480) 18. Finally, single-cell transcriptome data from the motor cortices of LPS-challenged and control mice were downloaded from the GEO (GSE211099) 19.

### Animals

C57BL/6 mice were obtained from the Southern Medical University Animal Center and housed under standard laboratory conditions in well-ventilated cages under a 12:12 h light-dark cycle with *ad libitum* access to food and water. Male mice aged 12-16 weeks were used unless otherwise stated.

### Mouse models of endotoxemia and sepsis

Mouse experiments mainly involved LPS and CLP models to simulate sepsis. The LPS administration model simulated an acute systemic inflammatory response through bacterial endotoxin stimulation, whereas the CLP model induced the complex pathological process of sepsis by surgically causing polymicrobial infection. C57BL/6 mice were randomly divided into experimental groups and received a single intraperitoneal injection of Dulbecco's phosphate-buffered saline (DPBS; #C14190500BT; Gibco, Billings, MT, USA) or LPS (isotype O111:B4; Sigma-Aldrich, St. Louis, MO, USA) dissolved in DPBS at 10 mg/kg body weight. A therapeutic model of VPA following the LPS challenge was used to investigate brain immunomodulation during neuroinflammation. In the experimental model, Valproic acid sodium (HY-10585A; MedChemExpress, Brunswick, NJ, USA) was administered intraperitoneally at a dose of 300 mg/kg body weight 30 min after the initial LPS injection. Subsequently, all mice were euthanized 24 h after the injection of DPBS or LPS (specific euthanasia time points are indicated in the corresponding figure legends) and their brain tissues were collected. CLP was performed as previously described 20. Briefly, the mice were anesthetized and subjected to a 1 cm midline laparotomy to expose the cecum. Approximately 50% of the cecum was ligated and punctured using a 21-gauge needle. A small amount of fecal matter was extracted from the punctured site. The cecum was replaced with the peritoneal cavity, and the abdominal incision was closed. To resuscitate the animals, 1 mL saline was injected subcutaneously. Mice were temporarily placed on a heating pad for recovery.

### Library preparation and RNA sequencing

Total RNA was extracted using a TRIZOL kit (Invitrogen, Carlsbad, CA, USA) according to the manufacturer's protocol. Subsequently, qualified RNA was reverse transcribed using SMARTScribe^TM^ reverse transcriptase (Clontech, Mountain View, CA, USA) to generate 5′-Rapid Amplification of cDNA Ends (RACE) cDNA for high-throughput sequencing. cDNA was purified using a MinElute PCR Purification Kit (Qiagen, Hilden, Germany), cDNA libraries were generated from total RNA, and cDNA libraries were sequenced using an Illumina HiSeq 2000.

### RNA-seq data alignment and quantification

The quality of the FASTQ files was assessed using FastQC and the reads were trimmed using TrimGalore. RiboDetector 21 was used to remove rRNA sequences using default settings. The filtered reads were aligned to the GRCm38 reference genome using HISAT2 22, followed by transcript assembly and quantification using feature counts 23. The expression values are expressed as counts per million (CPM).

### Cytokine detection

Fresh mouse brain tissues were removed, placed in ice-cold radioimmunoprecipitation assay (RIPA) buffer (#E121-01; GenStar, Beijing, China) containing a protease inhibitor cocktail (#4693159001; Merck, Darmstadt, Germany), and disrupted by sonication. Cytokine levels in the brain homogenates were measured using Luminex xMAP technology with a commercial kit (#PPX-08-MX2W9VA, ProcartaPlex; Thermo Fisher Scientific, Waltham, MA, USA) following the manufacturer's instructions. Cytokine levels in the brain homogenates were further analyzed using a mouse XL cytokine array (#ARY028; R&D Systems, Minneapolis, MN, USA) following the manufacturer's guidelines. Six biological samples from the wild-type (WT), LPS, or LPS+VPA groups were combined and tested separately, with a final volume of 100 µL (200 µg) for each array membrane.

### Statistical analysis

Data are expressed as the mean ± standard error of the mean (SEM). All statistical analyses (excluding RNA-seq data) were performed using GraphPad Prism version 7 (GraphPad Software, San Diego, CA, USA). Survival analysis was performed and visualized using the R packages survminer 24 and survival 25. The sample sizes and statistical testing methods used in each experiment are described in the figure legends.

### Development of DeconvCellLink R Package

DeconvCellLink is an innovative method for studying cell-cell communication by analyzing bulk RNA-seq data. A semi-supervised deconvolution algorithm 26 was used to analyze mouse RNA-seq data, allowing the estimation of cell type proportions and identification of potential markers for each cell type 27. Enrichment analysis was subsequently performed on genes that showed differential expression, as well as on potential cell markers. This analysis provides insights into the distinct biological functions and roles of different cell types. To convert our enrichment findings into a cellular communication network, we employed the Bayesian network inference technique 28. This facilitated the identification of crucial cell types involved in these interactions and the mechanisms through which they interact. We used data from an extensive database of ligand-receptor interactions 29 to support our analysis, providing a robust basis for examining the cell communication network. This technique serves as a powerful tool for investigating complex biological systems and the cellular interactions driving these processes. Bulk RNA-seq data were used to extract the intricate details of the cellular communication process.

Additional methods are provided in the supporting methods.

## Results

### Disturbances in brain immune cell communication critical to SAE progression

**Figure 1:**
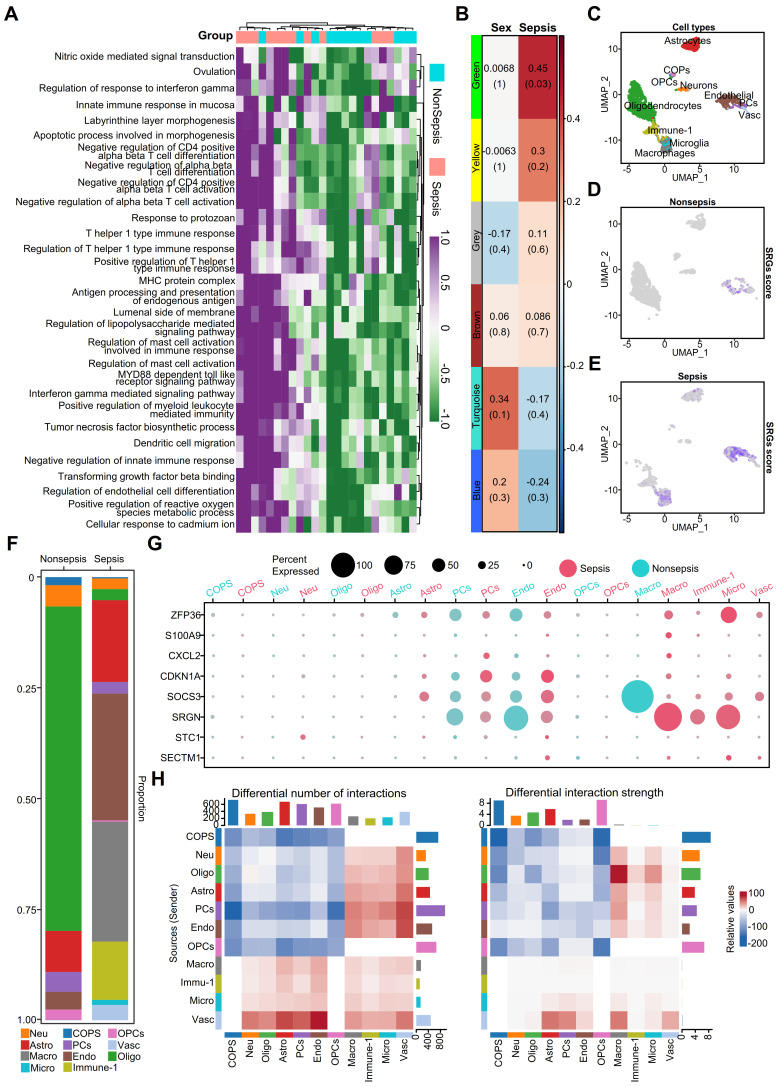
** Enhanced immune activity and cell type alterations in human septic brains. A.** Heatmap illustrating immune-related functions being significantly more active in septic brains compared to non-septic brains. **B.** Heatmap displaying gene co-expression modules associated with sex and sepsis. Values represent correlation coefficients, with corresponding *p*-values shown in parentheses. **C.** UMAP plot depicting eleven cell types identified from snRNA-seq data, including neurons, astrocytes, oligodendrocytes, COPs, OPCs, endothelial cells, macrophages, microglia, and other immune cells (Immune-1). **D.** UMAP plot showing the distribution of sepsis-related gene scores across cells in non-septic brain tissue. **E.** UMAP plot illustrating the distribution of sepsis-related gene scores across cells in septic brain tissue. **F.** Bar chart comparing the percentage of different cell types among the septic and non-septic brain tissues. **G.** Dot plot showing the expression of sepsis-related genes across various cell types in septic and non-septic brain tissues. **H.** Heatmap demonstrating the differences in the number and strength of cell-cell interactions between septic and non-septic brain tissues. UMAP, Uniform Manifold Approximation and Projection; COPs, committed oligodendrocyte precursors; OPCs, oligodendrocyte progenitor cells.

To examine the impact of sepsis on the human brain, we examined immune-related responses and cell type alterations using Gene Set Variation Analysis (GSVA). The results showed significantly increased immune-related activity in septic brains compared to that in non-septic controls. Key immune processes, such as the regulation of T-cell activation and myeloid leukocyte-mediated immunity, were elevated in the sepsis group **(Figure 1A)**. To assess patient heterogeneity, we analyzed the clinical characteristics of patients with (n = 12) and without sepsis (n = 12). The age distribution of the two groups was comparable (91.2 ± 5.6 years vs. 88.7 ± 9.4 years), but the gender composition differed (sepsis group: 4 males/8 females; non-sepsis group: 8 males/4 females). The distribution of underlying neurological diseases, such as dementia, was balanced between the two groups (three patients in each group), minimizing potential confounding factors.

Based on these clinical characteristics, we performed a weighted gene co-expression network analysis (WGCNA), which identified five co-expression gene modules: green, yellow, brown, turquoise, and blue, while the gray module included non-co-expressed genes **(Figure 1B)**. The green module was significantly positively correlated with the pathological process of sepsis **(Figure 1B)**. Given the similarity in age between groups, we did not include age as a trait but incorporated gender as a feature to identify sex-related gene modules. Genes with high module characteristic gene connectivity (kME ≥ 0.8) in the green module were defined as sepsis-related genes (SRGs) **(Table S2)**.

The functions of SRGs in the septic brain were revealed by the Gene Ontology (GO) enrichment analysis, which demonstrated correlations between immune regulatory mechanisms and endothelial cell disorders **(Figure S2A)**. Protein interaction network analysis revealed interleukin (IL) 1 beta (β) as central player in sepsis-related brain alterations **(Figure S2B)**, supporting previous research linking IL1β to cognitive dysfunction 30. GeneWalk analysis identified potential regulatory genes involved in sepsis, including *IL1B*, *CDKN1A*, CDK2, *CSF3*, and *IL1R2*, which were included among the SRGs **(Figure S3A, Table S3)**. Gene moonlighting analysis revealed that none of the differentially expressed genes (DEGs) were multifunctional, indicating their conserved functions in acute septic brain inflammation **(Figure S3B)**.

Next, using snRNA-seq data, we identified 11 SRG-associated cell types **(Figure 1C)**: neurons, astrocytes, oligodendrocytes, committed oligodendrocyte precursors (COPs), oligodendrocyte progenitor cells (OPCs), endothelial cells, macrophages, microglia, and immune cells (Immune-1). The proportion of oligodendrocytes was decreased in the septic brain, indicating demyelination injury **(Figure 1F)**. However, immune-related cells, including macrophages, microglia, and Immune-1 increased in the septic brain, indicating an elevated inflammatory response **(Figure 1F)**. Additionally, astrocytes and endothelial cells increased in the septic brain **(Figure 1F)**. We calculated a score based on SRGs expression **(Figure 1D-E)** and found that these genes were predominantly associated with macrophages, microglia, astrocytes, and endothelial cells in the sepsis group, indicating their role in sepsis progression. Dot plots show the differential expression of some SRGs in septic and non-septic brains, particularly in immune-associated and endothelial cells **(Figure 1G)**. However, SRGs such as *S100A9*, *CXCL2*, *STC1*, and *SECTM1* were less expressed in both the septic and non-septic groups **(Figure 1G)**. To understand the microenvironment in the septic brain, we used CellChat to compare intercellular communication between septic and non-septic brains. Cell-cell communication increased within immune cells and between immune cells and other cell types in the septic group **(Figure 1H)**, indicating an enhanced immune response. In contrast, the strength of cell-cell communication and interactions between non-immune cells was reduced, indicating disrupted physiological functions in the septic brain. These findings emphasize the importance of immune and endothelial cell disorders in sepsis-induced brain damage.

### The cellular changes in SAE exhibit consistency between humans and mice

**Figure 2:**
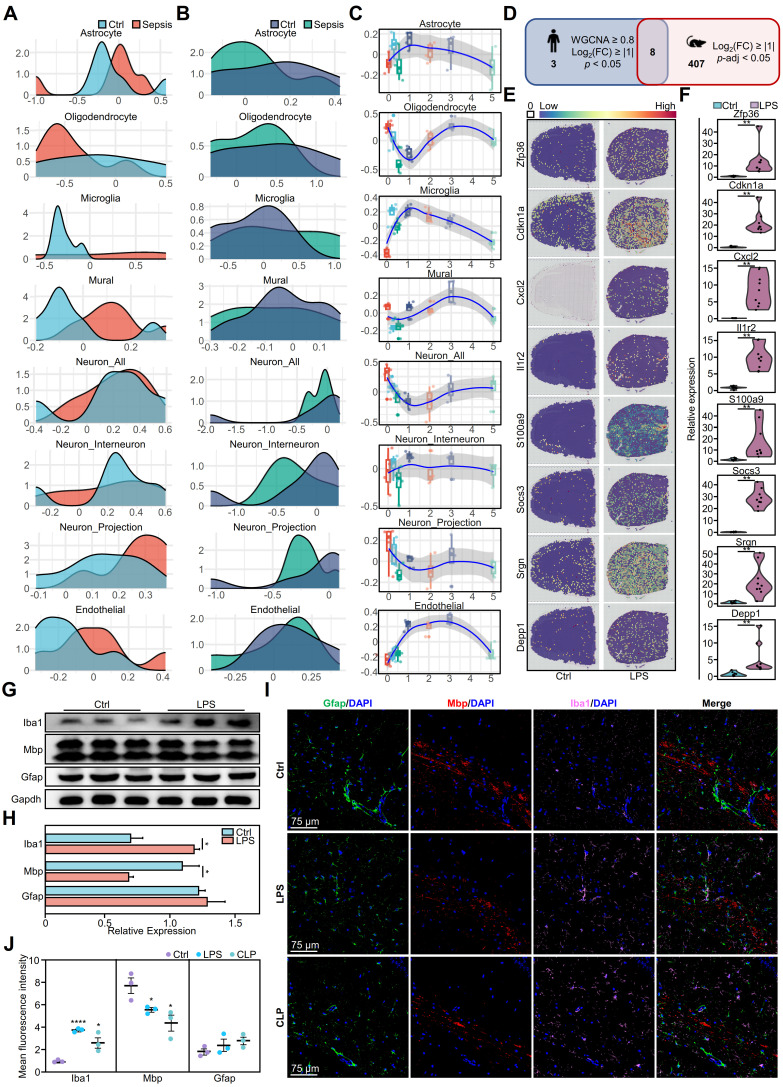
** Impact of systemic inflammation on brain cells and related gene expression. A-B.** Density plots showing the normalized abundance distribution of eight major brain cell types in the cortex **(A)** and hippocampus **(B)** of sepsis (red/dark blue) and non-sepsis (light blue/green) human brain samples (*n* = 12 per group). The x-axis represents the normalized abundance value, and the y-axis represents the probability density. Changes in the distribution curves indicate changes in the abundance of cell types under different conditions. **C.** Box plots showing the temporal changes of eight brain cell types in mouse brains following LPS challenge. **D.** Venn diagram depicting SRGs shared between humans and mice. **E.** Distribution of expression for eight sepsis-related differentially expressed genes in control and LPS-challenged mouse brains. **F.** Violin plots showing qPCR expression differences of eight representative sepsis-related genes in LPS-challenged and control mouse brains. Each group had nine replicates. Statistical significance was calculated using the Mann-Whitney test, **p < 0.01. **G.** Representative protein expression in LPS-induced mouse brains as detected by western blot. **H.** Histograms of the western blot results show statistically significant differences in cytokine levels between LPS and control groups over time. Statistical significance was determined using unpaired *t*-test, *n* = 3; *p < 0.05. **I.** Immunofluorescence staining showing the distribution of oligodendrocytes (red), microglia (pink), and astrocytes (green) in the hippocampi of mice 24 h after DPBS, CLP modeling, or LPS challenge. Blue represents DAPI staining. **J.** Statistical analysis of whole-brain mean fluorescence intensity in control, LPS-challenged and CLP-induced mice. Statistical significance was calculated by Mann-Whitney test, *p < 0.05, ****p < 0.0001. LPS, lipopolysaccharide; SRGs, sepsis-related genes; qPCR, quantitative polymerase chain reaction; DAPI, 4',6-diamidino-2-phenylindole; CLP, cecal ligation and puncture.

Deconvolution analysis was used to compare the distribution of the eight major cell types in different brain regions, providing insight into the microenvironmental changes in the septic brain. In the cerebral cortex, astrocytes, parietal cells, neurite neurons, and endothelial cells in the sepsis group showed an increasing trend, suggesting that these cells may have undergone proliferation or increased activity **(Figure 2A)**. Cortical microglia were predominantly distributed in the negative range in the control group, whereas the sepsis group exhibited high heterogeneity; however, the overall distribution shifted toward positive values, suggesting an increasing trend in microglia.

In the hippocampus, neurons (including all neurons, interneurons, and projection neurons) exhibited more pronounced changes, indicating that this region was more affected during sepsis **(Figure 2B)**. Similarly, the distribution of the hippocampal microglia showed an increasing trend. Notably, oligodendrocytes showed a consistent decrease in all brain regions studied, suggesting that sepsis-induced brain nerve damage is characterized by a widespread distribution **(Figure 2A-B)**.

Furthermore, to explore the neuropathological alterations in sepsis, we used time-series brain data from mice with endotoxemia from the public dataset 16 to analyze time-dependent dynamic changes in cell types **(Figure 2C)**. During the initial period (0-1 days), the number of various cell types fluctuated, indicating a chaotic brain microenvironment. Astrocytes and microglia initially exhibited an abrupt increase, reaching their highest point on day 1, and subsequently decreased gradually. Notably, endothelial cells showed no significant alterations initially, but quickly increased on day 1. Oligodendrocytes exhibited a rapid initial decrease, followed by a return to peak levels on day 3. Neurons initially exhibited a rapid decrease, followed by a gradual increase, whereas interneurons, projection neurons, and mural cells maintained a stable state.

We performed a comparative analysis of murine models **(Figure 2C)** and human data **(Figure 2A-B)**, which revealed that oligodendrocytes, microglia, and endothelial cells in mice displayed more pronounced early alterations, whereas late-stage mouse models and human septic brain demonstrated more moderate changes. Human neurons exhibited substantial disparities between the sepsis and control groups, whereas mouse neurons exhibited temporal fluctuations, suggesting that neural cells were in a regulated state. These similar changes observed in humans and mice highlight the importance of mouse models in understanding the pathological mechanisms of sepsis-induced brain injury.

We performed a comparative analysis of gene expression in brain tissues from mice with endotoxemia and identified DEGs. These results were then compared with those of DEGs in the human brain during sepsis. The analysis showed that *ZFP36*, *CDKN1A*, *CXCL2*, *IL1R2*, *S100A9*, *SOCS3*, *SRGN*, and *DEPP1* exhibited similar expression patterns in humans and mice **(Figure 2D)**, indicating that these eight genes were conserved differentially expressed genes (CDEGs) in the inflammatory brain. We hypothesized that previously identified immune cell changes were associated with CDEGs. Thus, we investigated the ARCHS4 database and found that CDEGs were associated with immune-related disorders in humans and immune dysfunction phenotypes in mice, which verified and strengthened our hypothesis **(Table S4-S11)**. Subsequently, we analyzed the expression distribution of these CDEGs in both humans and mice. The genes in normal mouse brains exhibited a uniform distribution **(Figure S4A)**. However, in LPS- challenged mice, the expression of these genes was significantly upregulated and evenly distributed throughout various regions of the brain **(Figure 2E, Figure S4B)**. Quantitative polymerase chain reaction (qPCR) revealed a significant increase in gene expression in the LPS group **(Figure 2F)**. Further analysis showed increased expression of these genes in almost all brain regions in the sepsis group **(Figure S4C)**. A similar pattern of gene expression upregulation was also observed in human brain regions under septic conditions **(Figure S4D-E)**.

Additionally, we verified the presence of well-established indicators in mouse models of endotoxemia. Western blotting analysis of mouse models of endotoxemia showed an increase in the microglial marker Iba1 and a decrease in the oligodendrocyte marker Mbp, but no change in the astrocyte marker Gfap **(Figure 2G-H)**. Immunofluorescence analysis of the hippocampus showed that Iba1 was significantly increased and Mbp was significantly decreased in the LPS and CLP groups compared to the control group **(Figure 2I-J)**. However, subregional statistics demonstrated alterations in the expression of Iba1, Mbp, and Gfap in 11 different brain regions in both the LPS and CLP groups compared to those in the control group **(Figure S5A-B)**. Both groups showed alterations in glial cell markers, with variations observed across regions and cell types. The LPS group exhibited higher levels of microglial and astrocytic activation in various brain regions, including the cerebral cortex, basal ganglia, cerebellum, thalamus, pons, and hypothalamus **(Figure S5C-M)**. In contrast, the CLP group exhibited a significant increase in Gfap levels, particularly in the hippocampus. The expression of the oligodendrocyte marker Mbp exhibited a declining pattern in both models, with a more pronounced decrease in the CLP group **(Figure S5C-M)**.

### Myeloid cells participate in regulating the brain microenvironment during systemic inflammation

**Figure 3:**
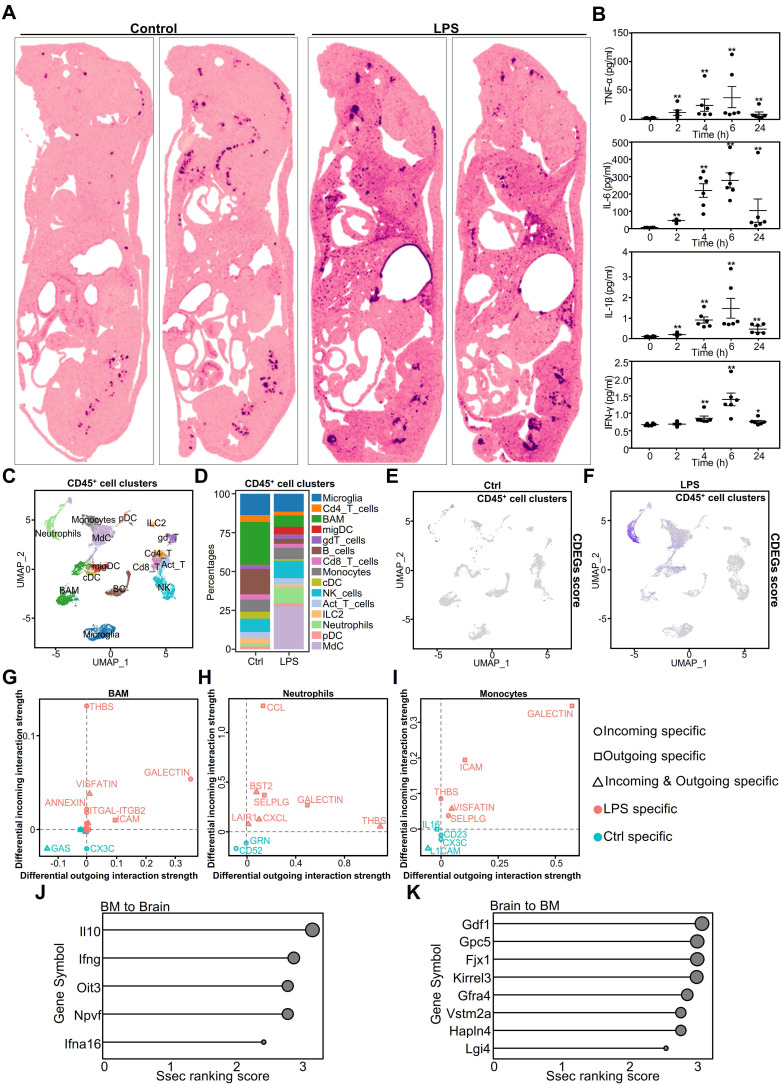
** Multifaceted analysis of systemic inflammation effects on mouse brain and immune system. A.** Whole-mouse spatial transcriptomics analysis of CDEGs scores overlaid on hematoxylin and eosin (H&E) staining. **B.** Cytokine expression in the brains of mice challenged with LPS for 0, 2, 4, 6, and 24 h, as detected using a multiplex immunoassay. Statistically significant differences in cytokine levels between the LPS and control groups over time are indicated by black asterisks. (Mann-Whitney, *n* = 6; *p < 0.05, **p < 0.01). **C.** Single-cell transcriptome analysis of CD45^+^ cell clusters isolated from the whole brain (parenchyma and border regions) of mice after PBS or LPS challenge. CD45^+^ cell clusters were visualized by UMAP with different colors. **D.** Proportion of 15 CD45^+^ immune cells in the whole brain (parenchyma and border regions) of mice post PBS or LPS challenge. **E.** UMAP plot of CD45^+^ brain cells from mice after PBS challenge. The expression levels of genes in the CDEGs were visualized using blue intensity.** F.** UMAP plot of CD45^+^ brain cells from mice after LPS challenge. The CDEGs score was visualized as described above. **G-I.** Scatter plot showing differences in outgoing (or incoming) signaling between representative CD45^+^ cells in the PBS or LPS-challenged groups. **J-K.** Lollipop plots showing tissue-specific regulatory factors between bone marrow (BM) and brain. **(J)** Regulatory factors from BM to brain. **(K)** Regulatory factors from the brain to BM. Strength of cross-tissue predictions for endocrine circuits (Ssec) ranking score (0-3) indicates the relative importance of each factor in the tissue-specific regulatory network. Dot sizes correspond to the Ssec ranking scores. UMAP, Uniform Manifold Approximation and Projection; ILC, innate lymphoid cells; pDC, plasmacytoid dendritic cells; migDC, migratory dendritic cells; LPS, lipopolysaccharide; CDEGs, conserved differentially expressed genes; PBS; phosphate-buffered saline.

Sepsis causes widespread inflammation in the brain, and CDEGs are associated with septic brain conditions; therefore, we hypothesized that they may be related to other organs or tissues. Using transcriptome data from mouse array sequencing, we conducted gene scoring and projection analyses **(Figure 3A, Figure S6)**.

Our findings indicated that CDEGs predominantly co-localized with the bone marrow in the control group. In the LPS group, CDEGs were localized to the bone marrow spinal cord, liver, lungs, and brain. This suggests that CDEGs are associated with myeloid cells. To investigate the presence of myeloid-related cytokines in the brain, we established an LPS-challenged model and used the Luminex platform to measure four well-defined inflammatory factors associated with myeloid cells in the mouse brain **(Figure 3B)**. The results showed a dynamic pattern of an initial surge followed by a decline, indicating the infiltration of myeloid cells into the brain in response to systemic inflammation.

We further analyzed single-cell (sc)RNA-seq data from whole-brain parenchymal cells or CD45^+^ cells to identify CDEG-associated cell types. Uniform Manifold Approximation and Projection (UMAP) plots of brain parenchymal cells showed neurons, astrocytes, oligodendrocytes, microglia, and endothelial cells **(Figure S7A)**. *Zfp36*, *Cxcl2*, *Socs3*, and *Srgn* were predominantly expressed in the microglia and endothelial cells **(Figure S7B)**. *Cdkn1a* was primarily expressed in endothelial cells and oligodendrocytes, and *Depp1* in endothelial cells. *Il1r2* and *S100a9* exhibited minimal expression in brain parenchymal cells, indicating their predominant expression in immune cells.

The UMAP analysis identified 15 CD45^+^ immune cell types **(Figure 3C)**. In the LPS- challenged group, the proportions of monocyte-derived neutrophils and myeloid-derived cells (MdC) increased significantly **(Figure 3D)**. Although the proportion of T cells changed minimally **(Figure 3D)**, their signaling pathways were significantly altered, particularly the enhancement of programmed death ligand 1 (PD-L1) signaling **(Figure S9A)**, suggesting that T cell function may be inhibited. CDEGs in immune cells exhibited diverse expression patterns. For example, *Zfp36* and *Cdkn1a* were upregulated in immune cells, particularly neutrophils and monocytes. *Cxcl2*, *Il1r2*, and *S100a9* were predominantly expressed in neutrophils **(Figure S8B)**. Through analysis of the InnateDB 31 database, we found that three genes, *S100a9*, *Cxcl2*, and *Il1r2*, are involved in the innate immune response in both humans and mice. *Socs3* is expressed in most immune cells, whereas *Srgn* is expressed in both lymphoid and myeloid cells, indicating that it is involved in immune cell activation. *Depp1* showed limited expression in the immune cells **(Figure S8B)**. Our results suggest that CDEGs in the brain are primarily associated with myeloid and endothelial cells. When analyzing the CDEGs scores in immune cells, it was observed that they were particularly elevated in monocyte-derived cells and neutrophils **(Figure 3E-F)**. This suggests that CDEGs indicate endothelial cell activation and neutrophil infiltration. These findings suggest that systemic inflammation affects the genes that disrupt the immune microenvironment of the brain. We further analyzed the functions of myeloid cells that were significantly altered in sepsis, including neutrophils, monocytes, monocyte-derived cells (MdCs), border-associated macrophages (BAMs), and microglia **(Figure S8C)**. After the LPS challenge, neutrophils showed the strongest inflammatory response, followed by BAMs and microglia. Notably, monocytes and MdCs had reduced inflammatory scores, showing anti-inflammatory properties, whereas microglia exhibited a pro-inflammatory phenotype. The increased functional scores of neutrophils and BAMs suggested that these cells may infiltrate the central nervous system through a chemokine-mediated migration mechanism and drive a sustained inflammatory cascade in the microglia.

We evaluated the communication between 13 immune cell types using CellChat. Innate immune cells, such as monocytes, BAMs, and neutrophils, generated more LPS-specific signals than adaptive immune cells **(Figure 3G-I)**. Most immune cells exhibited thrombospondin (THBS)-related signaling **(Figure S9A)**, which modulates innate immunity 32. Neutrophils send and receive signals, suggesting that they may also regulate other cells. Notably, almost all T cells received PD-L1 signals in the LPS group **(Figure S9A)**, whereas myeloid cells, such as monocytes, neutrophils, and MdCs, exhibited increased *Cd274* expression in response to the LPS challenge **(Figure S9B)**.

T cells, innate-like lymphocytes, and natural killer (NK) cells produce interferon (IFN)-γ 33, which, as an immunomodulator, is crucial in sepsis 34. During the LPS challenge, NK cells primarily expressed IFN-γ. The expression of neutrophil interferon-gamma receptor 2 (IFNGR2) was elevated **(Figure S9B)**, suggesting that neutrophils are regulated by NK cells. Neutrophils generate PD-L1 in response to IFN-γ stimulation, thereby inhibiting the proliferation of adaptive immune cells such as CD4^+^ T, CD8^+^ T, gamma delta T, innate lymphoid, plasmacytoid dendritic, and migratory dendritic cells 35. These findings highlight the multifaceted role of neutrophils in modulating immune responses, particularly in sepsis and inflammation, such as during LPS challenge.

Moreover, we investigated the inter-tissue endocrine circuits between the brain and the bone marrow of mice challenged with LPS **(Figure 3J-K)**. The cytokines IL10 and IFNG, known for their involvement in immune balance and inflammation regulation, are involved in communication between the bone marrow and brain 36, 37. Glypican 5 (Gpc5) is associated with processes related to neuroinflammation 38, and Four-Jointed Box Kinase 1(Fjx1) is associated with infection 39.

These results highlight the complex interplay between the innate and adaptive immune responses, emphasizing the regulatory role of neutrophils. The dual interaction between the brain and bone marrow provides new and valuable insights into systemic inflammatory responses.

### Time-series data reveals dynamic communication patterns in mouse brains during systemic inflammation

We hypothesized that bone marrow-derived cells, particularly neutrophils, have a significant impact on brain neurons. However, current research techniques are insufficient to infer an association between the immune response in the brain and alterations in specific groups of cells. To address this issue, we developed a method, DeconvCellLink, which utilizes bulk RNA-seq to identify changes in the immune microenvironment of the brain.

**Figure 4:**
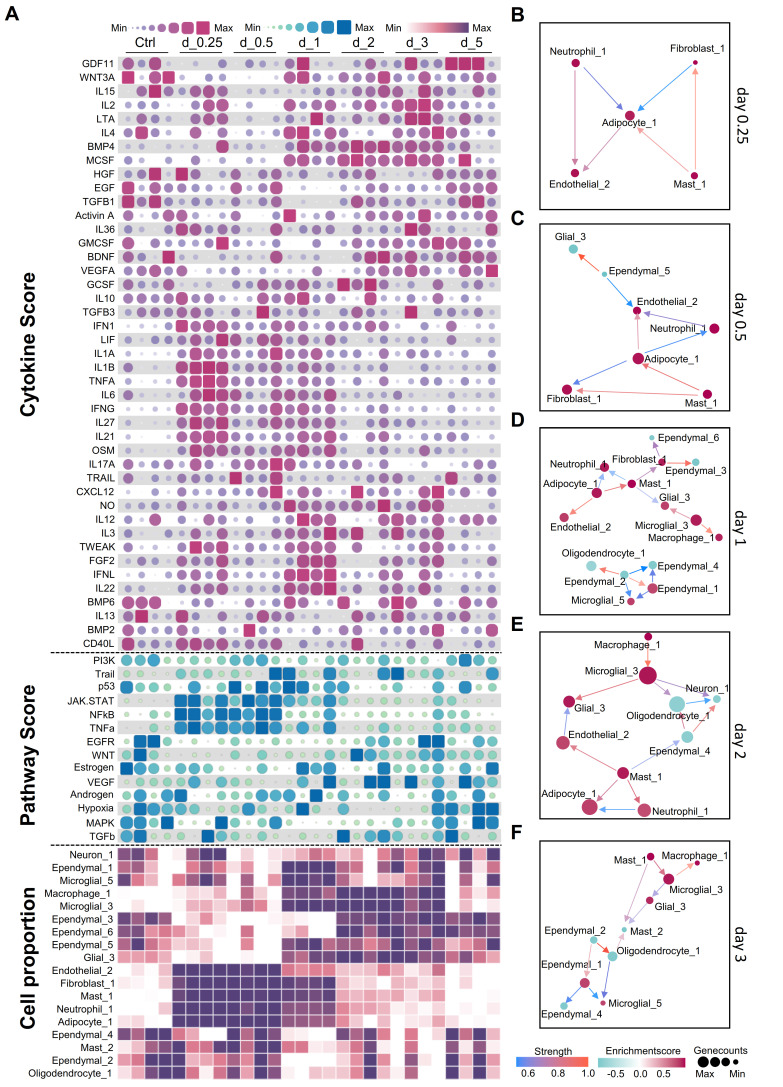
** Analysis of dynamic changes in the cerebral microenvironment of mice during systemic inflammation. A.** Heatmap showing temporal changes in cytokine (top), pathway activation (middle), and cell type activity scores (bottom) in the mouse brain after lipopolysaccharide (LPS) challenge. Color intensity represents relative expression levels or activity scores. Time points post-LPS challenge are indicated at the top of the heatmap. **B-F.** Cell-cell interaction networks at different time points following LPS challenge. Nodes represent different cell types, and edges indicate potential cell-cell interactions. Day 0.25 **(B)**; day 0.5 **(C)**; day 1 **(D)**; day 2 **(E)**; day 3 **(F)** post-LPS challenge.

During the early inflammation stage (0.25-1 day), pro-inflammatory cytokines such as IL-6, IL-1β, and tumor necrosis factor alpha (TNF-α), are significantly elevated. This increase correlates with the strong activation of the Janus kinase/signal transducers and activators of transcription (JAK-STAT), nuclear factor kappa B (NFκB), and TNF-α pathways **(Figure 4A)**, indicating that these cytokines are responsible for activating these pathways. We observed an increase in the number of endothelial cells (Endothelial_2), fibroblasts (Fibroblast_1), adipocyte-like cells (Adipocyte_1), and myeloid cells, including neutrophils and mast cells **(Figure 4A)**. Analysis of ligand-receptor interactions revealed significant interactions among neutrophil_1, adipocyte_1, endothelial_2, and Mast_1, indicating their crucial roles in the early inflammatory response **(Figure S10A)**. These cell types displayed significant interaction patterns, even during the early stages of inflammation **(Figure 4B-C)**. Notably, interactions involving IL-1 receptor antagonist (IL-1Ra, encoded by *Il1rn*) and IL-1 receptor type 1 (IL-1R1, encoded by *Il1r1*), as well as IL-1β (encoded by *Il1b*) and IL-1R1 (encoded by *Il1r1*) among the neutrophils and adipocyte-like cells highlight their crucial roles in regulating the inflammatory response **(Figure S10A)**. Moreover, the interactions observed between angiopoietin-like 4 (Angptl4) and cadherin 5 (Cdh5) in Adipocyte_1 and Endothelial_2 cells indicate their role in maintaining the inflammatory response **(Figure S10A-C)**.

The transition phase (1-2 days) marks a shift from inflammation to tissue repair. During this phase, pro-inflammatory cytokine levels decreased, whereas growth factors such as vascular endothelial growth factor A (VEGF-A), epidermal growth factor (EGF), transforming growth factor beta 1 (TGF-β1), and brain-derived neurotrophic factor (BDNF) increased **(Figure 4A)**. Similarly, the epidermal growth factor receptor (EGFR), vascular endothelial growth factor (VEGF), and Wnt signaling pathways were activated. On day 1, the cell interaction network became increasingly intricate, encompassing interactions between microglia, macrophages, adipocyte-like cells, and ependymal cells **(Figure 4D)**. On the second day, the network became less complex **(Figure 4E)**. Ligand-receptor interaction analysis identified Wnt3-Ror2 and Rspo2-Lgr6 interactions, indicating the involvement of Wnt signaling. Additionally, interactions such as Ncam1-Ncam1 and Fn1-Itgb8 suggest the initiation of the tissue repair process **(Figure S10C)**. On day 2, the interactions between Hmgb1 and Tlr2 and Nampt and Insr suggested that growth factors continued to influence the immune response and facilitate repair **(Figure S10D)**.

During the resolution and repair stage (2-5 days), inflammation subsided and repair processes dominated. TGF-β1 expression persists, and the activation of the Wnt signaling pathway continued. This stage was characterized by alterations in ependymal cell subtypes and an increase in glial cells, indicating that TGF-β1 and Wnt signaling drive ependymal layer modeling and glial cell normalization. On day 3, interactions were focused on macrophages, microglia, and ependymal cells **(Figure 4F)**. Notably, interactions such as Ncam1-Ncam1 and Cadm3-Cadm3 among ependymal cells indicate their involvement in tissue repair **(Figure S10E)**. By day 5, the cell interaction network had stabilized and could no longer generate new interactions, suggesting a return to homeostasis. These findings indicate that the timing of treatment plays an important role in the development of therapeutic strategies aimed at modulating immunity. Intervention for neuroinflammation is important during the early stages of inflammation.

### VPA reduces the effects of acute inflammation in the brain by modulating the immune microenvironment

Our investigation of treatments for SAE aimed to identify drugs with established anti-inflammatory properties that are currently being used in clinical settings for extended periods. Over the last five decades, VPA has gained recognition for its efficacy in managing neurological disorders. It is a multi-target drug that protects the nervous system and influences the immune microenvironment 40. Considering the proven effectiveness of VPA in treating organ injury caused by sepsis 41-44, we evaluated its potential as a therapy for LPS-induced brain inflammation.

**Figure 5:**
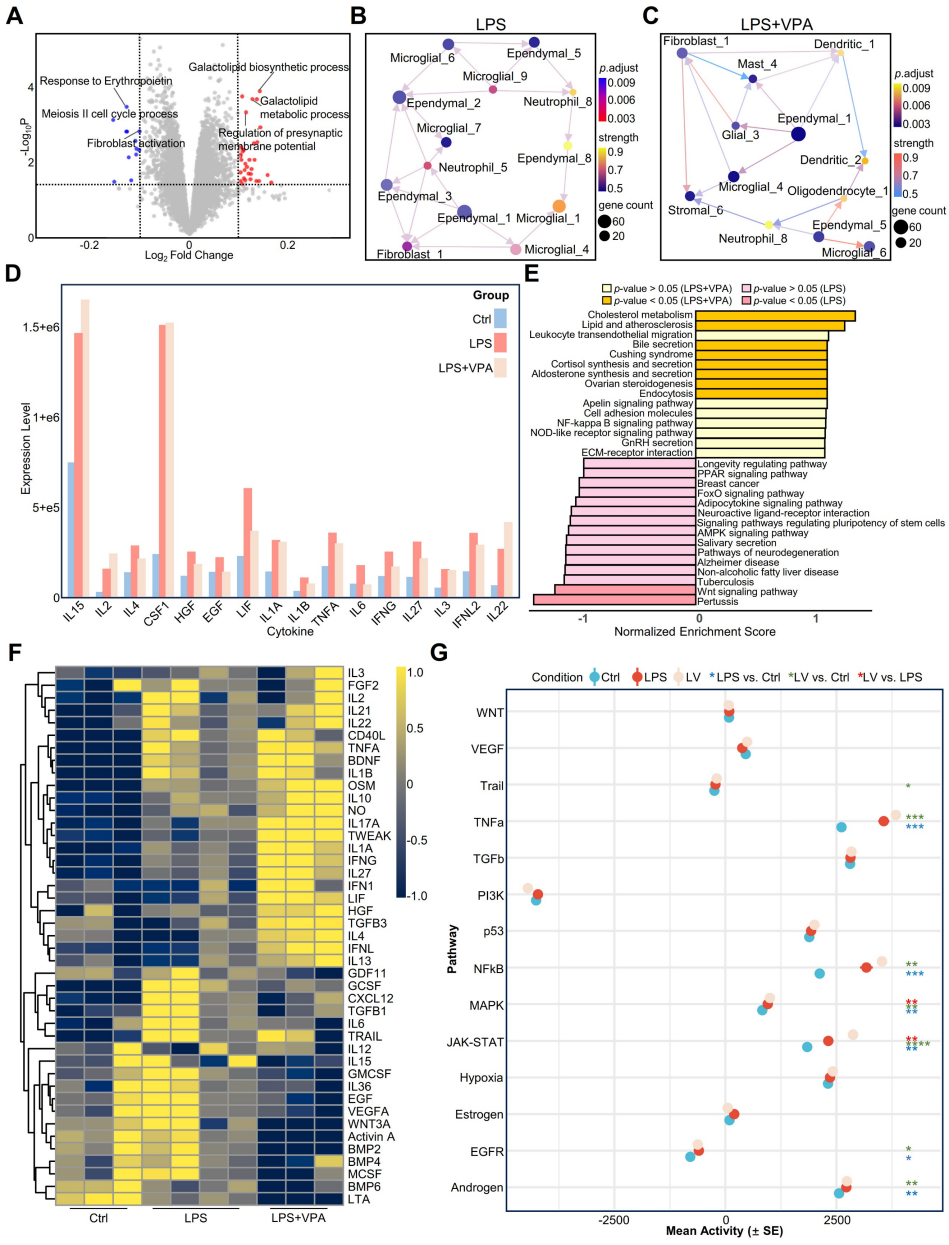
** VPA treatment modulates LPS-induced inflammatory responses in the mouse brain**.** A.** Volcano plot of differentially expressed genes between the LPS and LPS+VPA groups after GSVA analysis. Red and blue dots represent genes upregulated in LPS and LPS+VPA groups, respectively. **B-C.** Cell-cell interaction networks in LPS **(B)** and LPS+VPA **(C)** mouse brains. Nodes represent cell types; edges indicate interactions.** D.** Representative cytokine levels measured by cytokine array across the three groups. Different colors represent different groups. **E.** GSEA results comparing cytokine profiles between the LPS+VPA and LPS groups. Bars show normalized enrichment scores for KEGG pathways, with significantly enriched pathways highlighted. **F.** Heatmap of cytokine activity across all groups. Color intensity indicates relative cytokine activity levels under each condition. **G.** Mean activity of key signaling pathways in all conditions. Statistical significance was determined by ANOVA, *n* = 3-4; *p < 0.05, **p < 0.01, ***p < 0.001, ****p < 0.0001. VPA, valproic acid; LPS, lipopolysaccharide; GSVA, Gene Set Variation Analysis; GSEA, Gene Set Enrichment Analysis; KEGG, Kyoto Encyclopedia of Genes and Genomes; ANOVA, analysis of variance.

We employed GSVA to conduct a comparative analysis between the LPS and LPS+VPA groups **(Figure 5A)**. These findings indicate that the LPS group exhibited a stronger correlation with metabolic processes, whereas the VPA-treated group demonstrated a more association with cellular responses, specifically fibroblast activation. Subsequent analysis using DeconvCellLink showed that the LPS and LPS+VPA groups had a greater number of neutrophil subtypes and a smaller number of ependymal subtypes than the control group **(Figure S11A)**. In particular, the LPS group showed a higher proportion of neutrophil_1 and neutrophil_5, indicating notable immune activation signals **(Figure S11A)**. The LPS+VPA group showed a significant decrease in these subtypes, along with other neutrophil phenotypes that were primarily associated with metabolism and epigenetics. Notably, neutrophil_9 was associated with histone acetylation, an epigenetic effect of VPA 40, indicating that VPA influences neutrophil function via epigenetic modulation **(Figure S11A, Figure S12)**.

Using CDEGs to evaluate the LPS and LPS+VPA groups demonstrated that the expression levels of *S100a9* and *Il1r2*, which are mainly found in neutrophils, were not significantly different. However, *Cxcl2* expression increased following VPA treatment, suggesting a change in neutrophil function rather than a decrease in quantity **(Figure S11D)**. Moreover, the expression levels of *Depp1*, which is mainly observed in endothelial cells, and *Cdkn1a*, which has been identified in both endothelial cells and oligodendrocytes, significantly increased **(Figure S11D)**. This increase indicates improved integrity of oligodendrocytes and the BBB after VPA treatment.

Further analysis of the immune cell networks in the mouse brain **(Figure 5B-C)** showed increased interactions between microglia and neutrophils in the LPS group, whereas the LPS+VPA group demonstrated more extensive interactions between glial and parenchymal cells. Analysis of ligand-receptor interactions revealed that the LPS group exhibited a higher number of interactions involving microglia and neutrophils, resulting in the production of inflammation-related ligands such as Itgam-Itgb2 and Ceacam1-Havcr2. In contrast, the LPS+VPA group exhibited interactions mainly between fibroblasts and stromal cells, specifically Tgfb3-Itgb5, indicating the activation of the TGF-β signaling pathway **(Figure S11B-C)**.

The cytokine array results showed that the levels of the inflammatory cytokines IL1A, IL1B, and TNF-α were higher in both the LPS and LPS+VPA groups than in the controls, with the LPS+VPA group displaying lower levels than the LPS group **(Figure 5D, Figure S13)**. Furthermore, myeloperoxidase (MPO) levels increased in both the LPS and LPS+VPA groups. However, the C-reactive protein (CRP) levels were lower in the LPS+VPA group than in the LPS group **(Figure S13)**. This demonstrates that VPA has a favorable effect on reducing inflammation, although it does not prevent neutrophil infiltration. Gene Set Enrichment Analysis (GSEA) pathway analysis of cytokines that were differentially expressed between the LPS and LPS+VPA groups demonstrated that VPA was involved in the regulation of metabolism, endocrine functions, inflammation, and cell adhesion pathways. In contrast, LPS was primarily associated with inflammation, metabolic disorders, and neurodegenerative pathways **(Figure 5E)**.

Cytokine activity profiling showed no significant decrease in the activity of pro-inflammatory cytokines TNF-α and IL1B in the LPS+VPA group compared to that in the LPS group. However, IL6 and G-CSF activities decreased, suggesting a decrease in neutrophil-induced inflammation, although active macrophages and microglia were still present. In addition, IL-10 and BDNF levels were elevated in the LPS+VPA group, indicating the anti-inflammatory and neuroprotective properties of VPA **(Figure 5F)**. Pathway analysis showed that inflammation-related pathways, including TNF-α, NFκB, mitogen-activated protein kinase (MAPK), and JAK-STAT, were increased in both LPS and LPS+VPA groups compared to that in controls **(Figure 5G)**. The LPS+VPA group showed a more pronounced increase in the MAPK and JAK-STAT pathways, indicating activation of endothelial cells or the BBB following VPA treatment.

These findings indicate that VPA reduced the effects of LPS by regulating the immune microenvironment. Instead of directly opposing the effects of LPS, VPA modifies the inflammation-induced differentiation of immune cells, thereby decreasing brain inflammation and minimizing damage.

### VPA remodels the neuroimmune landscape in neuroinflammation

**Figure 6:**
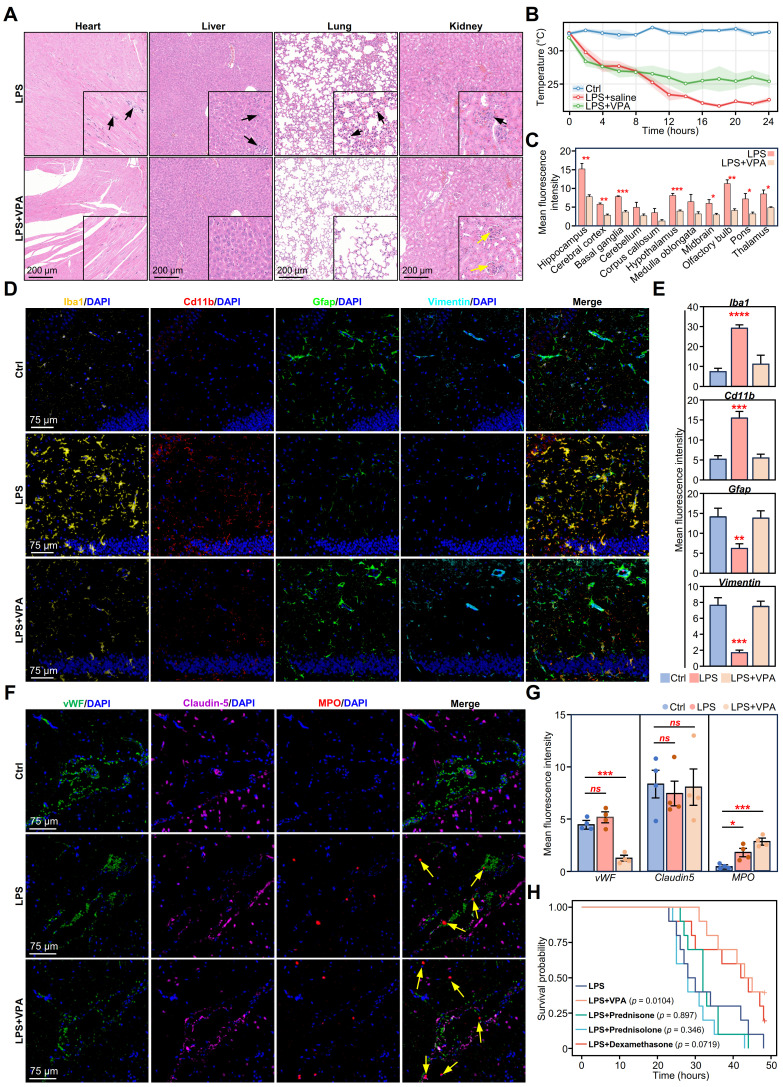
** Validation of VPA treatment effects on LPS-induced neuroinflammatory and BBB changes in mouse brain. A.** Representative H&E staining of heart, liver, lung, and kidney sections of mice 24 h after LPS and LPS+VPA. Black arrows indicate inflammatory cell infiltration and yellow arrows indicate glomeruli. Scale bars for main images and magnified insets are shown. **B.** Body temperature monitoring of mice injected with LPS or LPS+VPA over 24 h (*n* = 3 per group). **C.** Quantification of mean fluorescence intensity for Iba1 across different brain regions in LPS and LPS+VPA groups. Statistical significance determined by unpaired *t*-test, *n* = 3; *p < 0.05, **p < 0.01, ***p < 0.001. **D.** Representative immunofluorescence images showing Iba1 (yellow), Cd11b (red), Gfap (green), Vimentin (teal) and DAPI (blue) expression in the hippocampus of mice 24 h after DPBS, LPS, or LPS+VPA injections. **E.** Quantification of Iba1, Cd11b, Gfap, and Vimentin immunofluorescence intensity under different treatment conditions. Statistical significance determined by ANOVA, comparing LPS or LPS+VPA groups with the control group. *n* = 3; **p < 0.01, ***p < 0.001. **F.** Representative immunofluorescence images showing vWF (green), Claudin-5 (purple), MPO (red), and DAPI (blue) expression in the BBB of mice 24 h after DPBS, LPS, or LPS+VPA injections. Yellow arrows indicate neutrophil infiltration. **G.** Quantification of vWF, Claudin5, and MPO immunofluorescence intensity under different treatment conditions. Statistical significance determined by ANOVA, comparing LPS or LPS+VPA groups with the control group. *n* = 4; *p < 0.05, **p < 0.01, ***p < 0.001, ****p < 0.0001. **H.** Kaplan-Meier survival curves comparing LPS alone, LPS+VPA, and LPS+steroids (dexamethasone, prednisone, prednisolone) challenge groups (*n* = 10 per group). VPA, valproic acid; LPS, lipopolysaccharide; BBB, brain-blood barrier; H&E, hematoxylin and eosin; DAPI, 4',6-diamidino-2-phenylindole; DPBS, Dulbecco's phosphate-buffered saline; MPO, myeloperoxidase.

To evaluate the therapeutic potential of VPA in sepsis, we first evaluated its systemic effects. Hematoxylin and eosin (H&E) staining revealed a reduction in inflammatory infiltration and structural damage in the VPA-treated tissues compared to those in the LPS group, particularly in the lung, liver, kidney, and heart **(Figure 6A)**. Additionally, body temperature monitoring indicated that VPA treatment stabilized body temperature fluctuations induced by LPS, thereby demonstrating its protective effect against sepsis **(Figure 6B)**.

Subsequently, we assessed the impact of VPA on glial activation. Following VPA administration, Iba1 levels decreased in the hippocampi of LPS-challenged mice **(Figure S14A)**. Further examination of Iba1 expression across various brain regions revealed a significant reduction in Iba1 levels following VPA treatment, except in the cerebellum, corpus callosum, and medulla oblongata **(Figure 6C)**.

To further evaluate glial cell activation, we stained for microglial markers (Iba1 and Cd11b) and astrocyte markers (Gfap and Vimentin). The results showed that the expression of Cd11b and Iba1 in the hippocampus of the LPS+VPA group was significantly reduced. Conversely, the expression levels of Gfap and Vimentin remained the same as those in the control group but significantly decreased in the LPS group. These results indicate that VPA inhibited microglial activation while preserving astrocyte activity during the LPS challenge **(Figure 6D-E)**.

Furthermore, we evaluated the impact of VPA on neuronal activity and synaptic plasticity using the neuronal activation marker c-Fos and the synapse formation marker GAP43. Compared to the LPS group, c-Fos expression in the hippocampus was markedly elevated in the VPA-treated group, whereas GAP43 levels exhibited an increasing **(Figure S14B-C)**. These results suggest that VPA treatment may enhance neuronal function and facilitate synapse formation during neuroinflammation.

Next, we evaluated changes in the BBB. Claudin5, a key tight junction protein in the BBB 45, showed no changes among the different groups. This suggested that VPA had no significant effect on the tight junctions of the BBB **(Figure 6F-G)**. Notably, the endothelial cell marker vWF showed a downward trend in the LPS+VPA group compared to that in the LPS and control groups **(Figure 6F-G)**. This suggests that VPA may directly affect endothelial cell function rather than simply counteracting LPS-induced changes.

Furthermore, we investigated myeloperoxidase (MPO), an enzyme and functional marker specific to neutrophils 46. A significant increase in gene expression was observed in both the LPS and LPS+VPA groups **(Figure 6F-G; yellow arrow)**. This finding aligns with our previous CDEGs and DeconvCellLink analyses, confirming that VPA does not inhibit neutrophil infiltration **(Figure S11A, Figure S11D)**. In contrast, VPA has the potential to alter neutrophil function rather than reducing neutrophil numbers, highlighting its complex role in regulating neutrophil activity during neuroinflammation.

Given the widespread use of glucocorticoids in anti-inflammatory therapy, we evaluated the therapeutic efficacy of VPA and steroids (dexamethasone, prednisone, and prednisolone) in the LPS stimulation model. Survival analysis indicated that VPA treatment markedly enhanced the survival rate of LPS-stimulated mice, whereas none of the steroid treatments exhibited a significant survival benefit **(Figure 6H)**. Blood biochemical analysis further confirmed the organ-protective effects of VPA. Comparison to the LPS group, VPA treatment was associated with reduced levels of hepatic injury markers aspartate transaminase (AST) and alanine transaminase (ALT), as well as the renal function indicator creatinine (CREA). Both VPA and steroid treatments regulated triglyceride (TG) levels; however, none of the treatments prevented the LPS-induced reduction in blood glucose levels **(Figure S14D)**.

We further analyzed the effects of steroids on microglia and MPO levels. The results indicated an increase in Iba1 expression following treatment with all three glucocorticoids, with prednisone and prednisolone showing significant increases. Similarly, Cd11b levels increased following treatment with prednisone and prednisolone, whereas MPO did not show any significant changes **(Figure S14E-F)**. Analysis of the RNA-seq data revealed that prednisolone was the most effective at reducing inflammatory signaling pathways, whereas dexamethasone and prednisone had no significant effect on inflammation-related signaling pathways **(Figure S14G)**. These findings indicate that certain steroids can suppress inflammatory signals in the brain but are less effective in modulating the neural microenvironment.

To evaluate the therapeutic efficacy of VPA in a clinically relevant model, we investigated its role in CLP-induced sepsis. VPA treatment significantly improved the survival rate of CLP mice **(Figure S15A)**. Consistent with the findings from the LPS model, Iba1 levels in the hippocampi of post-CLP mice were markedly reduced following VPA treatment **(Figure S15B)**. Immunofluorescence analysis revealed that VPA markedly decreased the number of Iba1^+^ and Cd11b^+^ cells, indicating that microglial activation was suppressed. Additionally, the expression of Gfap and Vimentin in the VPA-treated group was significantly higher than that in the CLP group, indicating that VPA may safeguard astrocyte function **(Figure S15C-D)**. The results from the CLP model were consistent with those from the LPS model, further reinforcing the therapeutic potential of VPA in SAE.

These findings suggest that VPA can effectively regulate neuroinflammation by altering the balance of the neural microenvironment, rather than simply inhibiting inflammation.

## Discussion

This study systematically elucidated the cellular and molecular mechanisms underlying SAE, with a particular focus on the dynamic changes in the brain immune microenvironment under systemic inflammatory conditions. We observed consistent changes in the microglia, endothelial cells, and oligodendrocytes in the brains of humans and mice with systemic inflammation. The cross-species conservation of these changes highlights the central role of these cells in the neuroinflammatory response. Moreover, our findings highlight the functional diversity and regional specificity of glial cells, suggesting that understanding SAE requires a comprehensive view of the interactions between different glial cells.

The identified CDEGs are specifically expressed in endothelial, glial, and immune cells, and were upregulated in inflamed brain tissues in both humans and mice. This broad expression pattern suggests that sepsis-induced brain dysfunction is a global, rather than localized, response.

Among the cells associated with CDEGs, neutrophils play an important role. Their interactions with endothelial cells and microglia exacerbate inflammation. Recent studies have also confirmed the key role of neutrophils and emergency granulocyte production in the immunosuppressive process of sepsis 47. Different myeloid cell populations exhibit significant functional differences. Infiltrating monocytes and their derivatives show anti-inflammatory properties, whereas resident microglia exhibit proinflammatory phenotypes. This functional differentiation underscores the complex regulatory network of the immune response in the brain during sepsis. Our study reveals that the adaptive immune system undergoes important functional changes during sepsis. Although the number of T cells did not change significantly, their immunosuppressive signals were significantly enhanced, as shown by the activation of the PD-L1 signaling pathway and the upregulation of immunosuppressive markers. Previous studies have shown that the combined effects of adaptive immune dysfunction and changes in innate immune function increase the risk of secondary infections in patients with sepsis 48.

To elucidate the complex intercellular interactions, we developed the DeconvCellLink analysis tool, which infers intercellular communication networks from bulk RNA-seq data. This approach allowed us to identify dynamic changes in intercellular communication in the brain under inflammatory conditions. In the early stages of inflammation, endothelial cells, fibroblasts, and adipocyte-like cells are the first to become activated. Under inflammatory and oxidative stress conditions, glial cells accumulate lipid droplets 49, 50, and this metabolic reprogramming reflects the adaptive response of glial cells to systemic inflammation. As inflammation progresses, macrophages and microglia drive the tissue into a repair phase. Ultimately, ependymal and glial cells jointly participate in restoring homeostasis within the brain's immune microenvironment.

Recently, neuroimaging and biomarker studies have provided new insights into SAE. Magnetic resonance imaging showed that 70% of patients with sepsis had neurological complications 51, and diffusion-weighted imaging found that 50% of patients with sepsis in the intensive care unit (ICU) had brain damage 52. Blood biomarker studies have confirmed that neuron-specific markers (NSE, NfL) and glial-derived markers (S100β, Gfap) can effectively predict the onset and prognosis of SAE 53, 54. These advances highlight the importance of early diagnosis and intervention in SAE.

In terms of treatment strategies, VPA has demonstrated promising therapeutic potential and has entered clinical trials for ICU delirium (NCT02343575). VPA significantly improved the survival rate of mice in both LPS and CLP sepsis models, verifying the reliability of its therapeutic effects. Mechanistic studies have found that VPA exerts its effects by regulating rather than inhibiting neutrophils, and that the maintenance of regulated immune responses is clinically more valuable than extensive immunosuppression. Notably, a retrospective study of COVID-19 patients found that those who received VPA upon hospital admission had a lower risk of organ damage 55. This clinical observation aligns with our findings in animal models, further supporting its therapeutic potential in systemic inflammatory conditions. Beyond its role in neuroinflammation, VPA protects multiple important organs from inflammatory damage. Compared to other emerging strategies, such as immune checkpoint inhibitors and cytokine therapy 56, VPA may offer a more balanced therapeutic approach by modulating the immune response rather than suppressing it. Time-series analysis further revealed that early neuroprotective intervention is more effective than waiting for neurological symptoms to appear, providing a new perspective on immunomodulatory treatment for sepsis.

Understanding the mechanisms of neuroinflammation in sepsis may also provide insights into other acute neuroinflammatory conditions. Immune effector cell-associated neurotoxicity syndrome (ICANS), a neurological complication of CAR T-cell therapy, shares several key features with SAE, including BBB disruption 57, 58, cytokine storm 58, 59, and microglial activation 57, 58. However, important differences exist between the two diseases: SAE is an acute response to systemic infection, primarily involving myeloid cells, whereas ICANS usually occurs several days after CAR T-cell infusion and is dominated by T cell-mediated responses 60. Despite these mechanistic differences, the shared neuroinflammatory patterns suggest potential common therapeutic targets, particularly for improving BBB integrity and alleviating neuroinflammation. The efficacy of VPA in our SAE model suggests that it may have broad applications in treating inflammatory neurological diseases with similar pathological features.

In this study, an LPS-induced sepsis model was used to explore the neuropathological associated with SAE. This model offers several advantages for studying acute inflammatory responses. The LPS-induced inflammatory response is highly consistent and reproducible, providing a standardized platform for mechanistic research and drug evaluation. Additionally, the synchronized response allows precise tracking of immune cell dynamics and temporal changes in molecular pathways. Compared with live bacterial infection models, the LPS model more effectively focuses on inflammation-mediated organ dysfunction, making it an ideal research system for analyzing the molecular mechanisms of the brain's inflammatory microenvironment and neurological complications.

## Conclusion

In summary, this study revealed the key cellular and molecular mechanisms underlying sepsis-related brain dysfunction, highlighting that immune dysregulation in the inflamed brain is primarily driven by the overactivation of myeloid cells, especially neutrophils. Additionally, we confirmed the unique role of VPA in regulating neuroinflammation. VPA not only maintains the balance of the neuroimmune microenvironment but also protects multiple organ systems during systemic inflammation. Unlike traditional steroid therapy, which primarily suppresses the inflammatory response, VPA offers a more balanced approach by regulating rather than inhibiting the immune response. These findings provide valuable insights into early intervention strategies for SAE and contribute to the understanding of other acute neuroinflammatory diseases.

## Supplementary Material

Supplementary figures and tables, methods.
