# Supplementary material for: Modulation of brain immune microenvironment and cellular dynamics in systemic inflammation
Source: Theranostics. 2025 Apr 9;15(11):5153–71. doi: 10.7150/thno.107061 (PMC12036864; doi:10.7150/thno.107061)
Supplement: Supplementary file 1 — Supplementary figures and tables, methods. [file thnov15p5153s1.pdf]

SUPPORTING INFORMATION

Title: **Modulation of brain immune microenvironment and cellular dynamics in systemic inflammation**

Junhao Wang, Zhaoqian Zhong, Haihua Luo, Qizheng Han, Kan Wu, Aolin Jiang, Li Chen, Yanxia Gao, Yong Jiang

Correspondence:

Yong Jiang: jiang48231@163.com; Yanxia Gao, gaoyanxiazzu@163.com

The supplementary file contains:

Supplementary Figures 1 to 15

Supplementary Tables 1 to 11

Supplementary Methods

Supplementary Figures

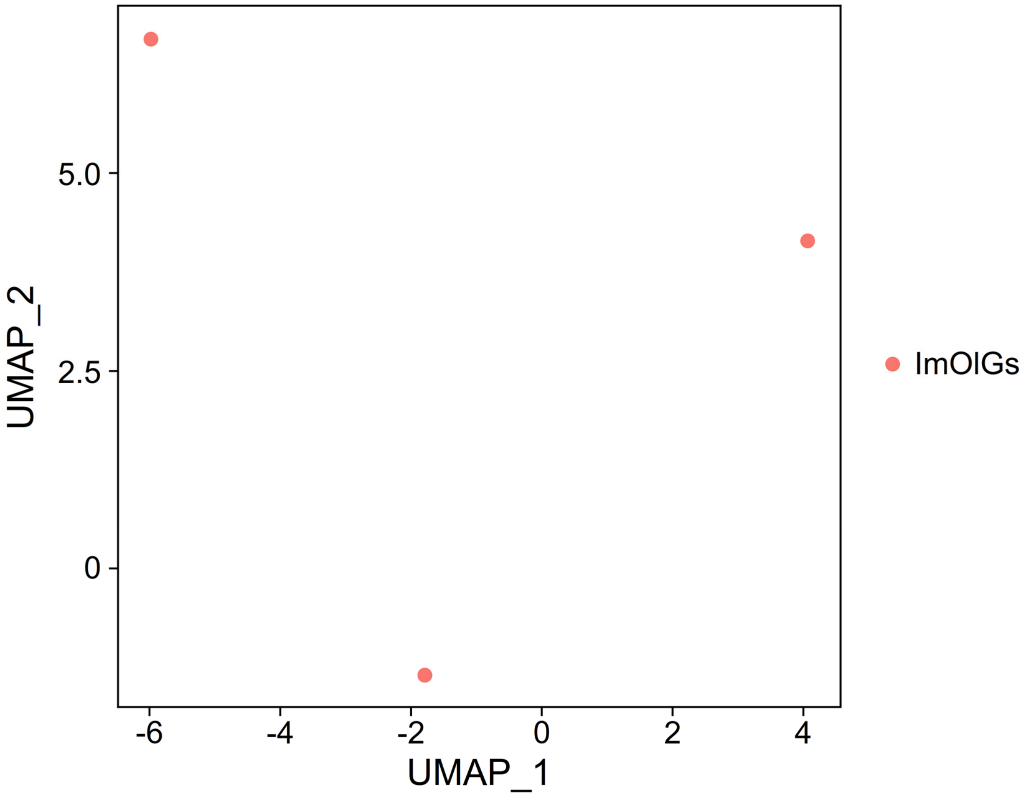

Supplementary Figure 1. Preprocessing of snRNA-seq data. Uniform Manifold Approximation and Projection (UMAP) plot showing the ImOlG (immune oligodendroglia) distribution.

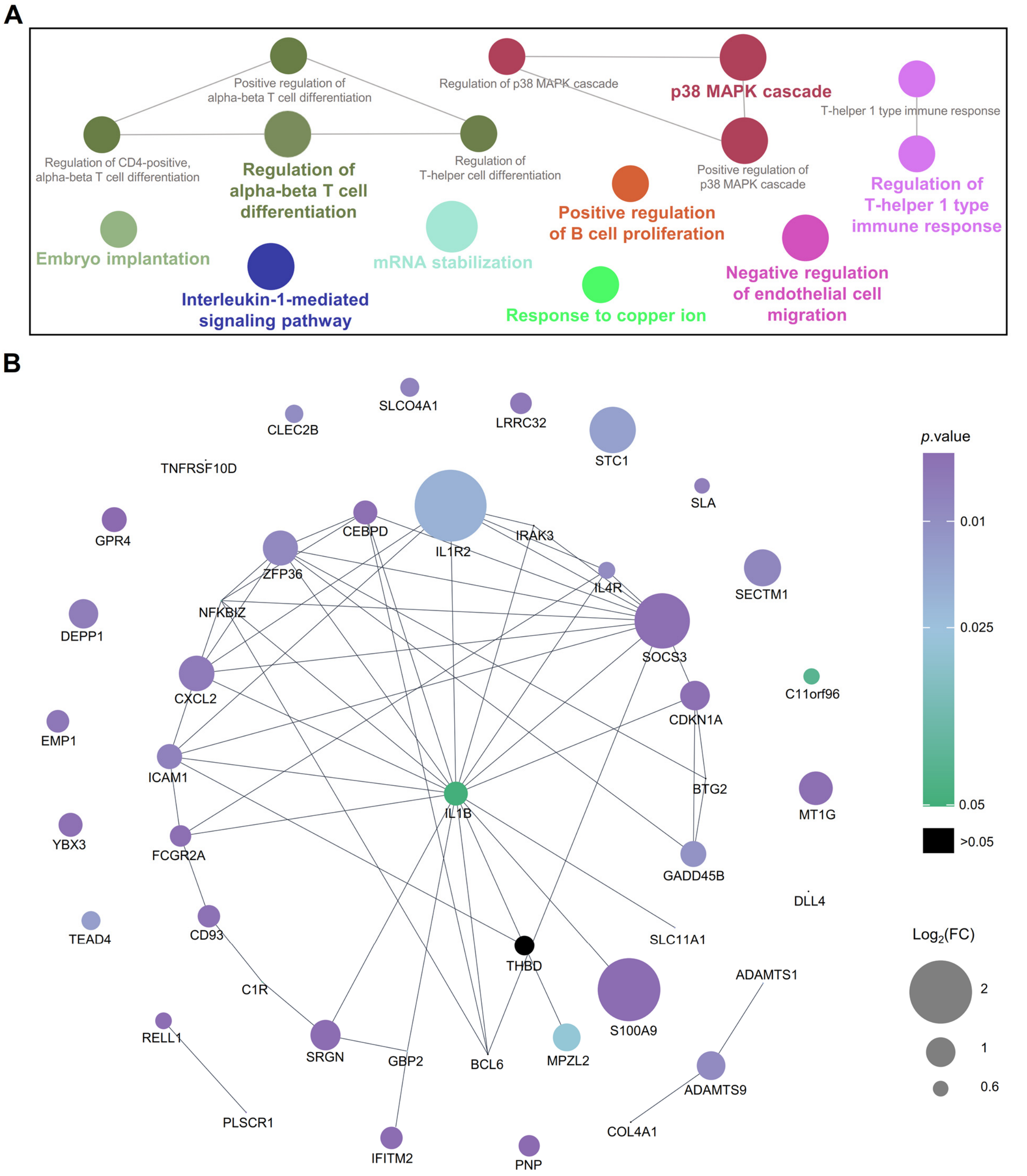

Supplementary Figure 2. Interactions of proteins encoded by sepsis-related genes. A. ClueGO enrichment analysis of the green module with statistical significance (*p* value < 0.05). **B.** Network showing the interactions of proteins encoded by sepsis-related genes from the STRING database. The dot size scales indicate the $\log_2$ fold changes of differentially expressed genes (DEGs) from septic and non-septic human brains, and the color scales of the *p* values indicate significant DEGs as determined by the analysis.

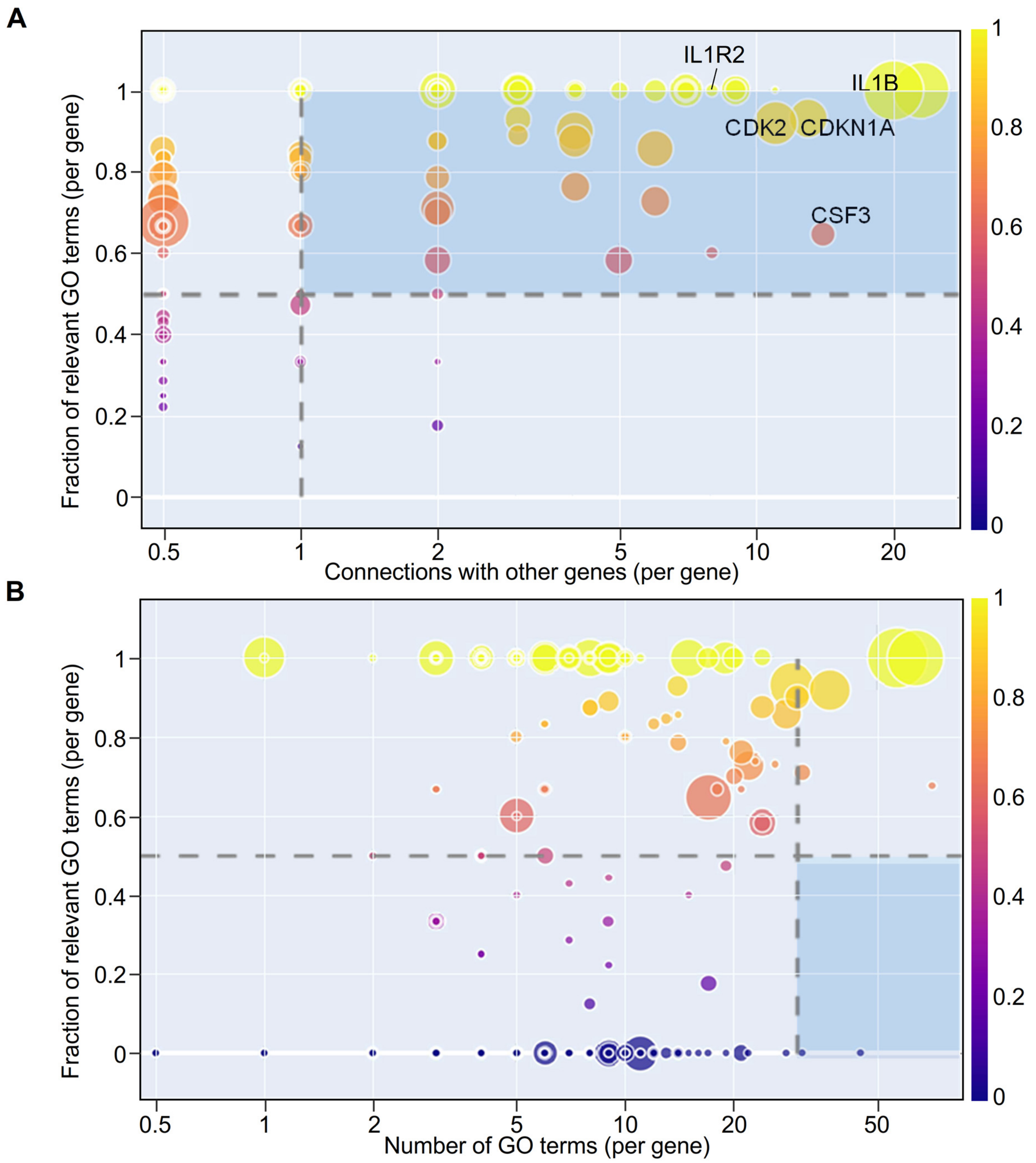

Supplementary Figure 3. Identify regulator genes of the human septic brain. A. Scatter plot with DEGs (|Log_2 fold change| >= 0.6) as data points showing the GeneWalk fraction of relevant GO terms over the total number of connected GO terms. The circle size of the scales indicates the differential expression significance strength (−Log_{10} *p*.adj), and the color hue indicates the fraction of relevant GO terms (per gene). Fifty-six genes in the blue-shaded area were identified as regulator genes (Supplementary Table 2). Some representative sepsis-related genes were identified as regulators. **B.** Scatter plot of DEGs as data points showing the GeneWalk fraction of relevant GO terms over the total number of GO term annotations. The circle size of the scales indicates the differential expression significance strength (−Log_{10} *p*.adj), and the color hue indicates the fraction of relevant GO terms (per gene). DEGs, differentially expressed genes; GO, gene ontology.

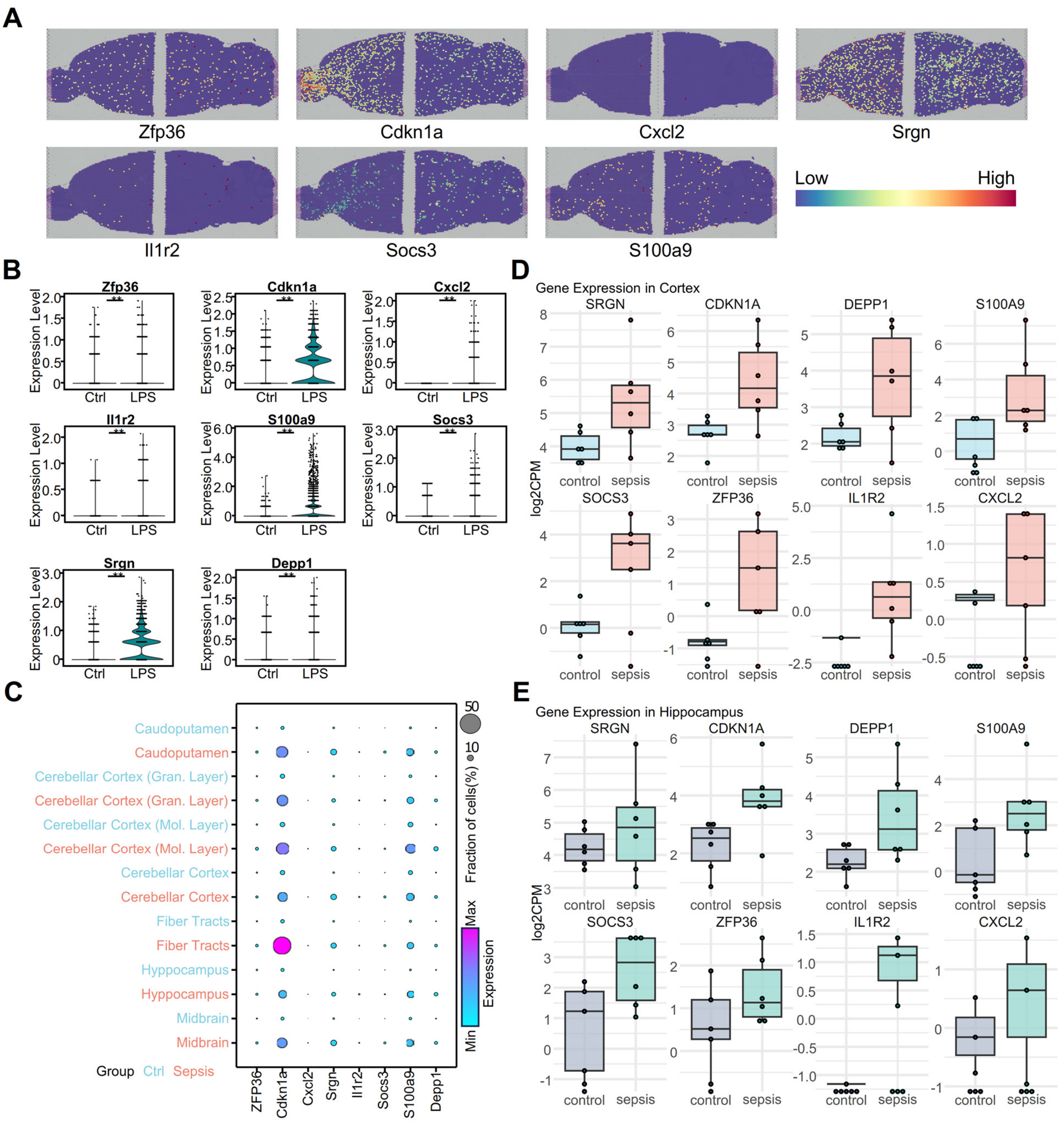

Supplementary Figure 4. Expression patterns of conserved differentially expressed genes (CDEGs) in mouse and human brains. A. Spatial expression of seven CDEGs in the mouse brain visualized using Allen Brain Map datasets from 10x Genomics Visium data. **B.** Violin plots illustrating the differential expression of CDEGs in lipopolysaccharide (LPS)-challenged and control mouse brains. Statistical significance was calculated using the Mann–Whitney test, **p*<0.05, ***p*<0.01. **C.** Dot plot showing the expression of sepsis-related (SR)-DEGs in various brain regions of LPS-challenged and control mice. **D.** Box plots displaying the expression differences of CDEGs in the cortex of non-septic and septic human brains. **E.** Box plots showing the expression differences of CDEGs in the hippocampus of non-septic and septic human brains.

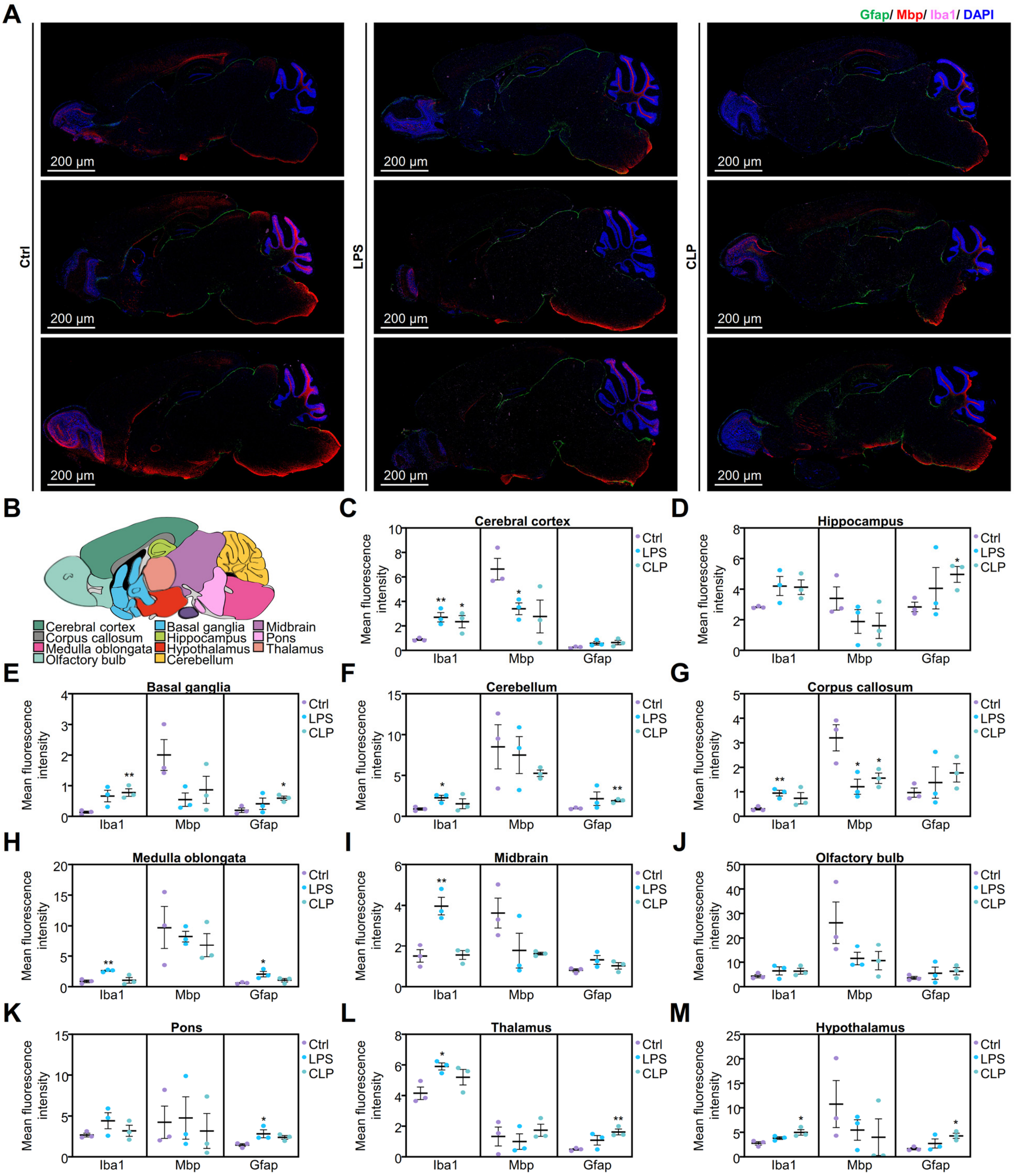

Supplementary Figure 5. Glial cell distribution and regional analysis in mouse brain under systemic inflammatory conditions. A. Immunofluorescence staining showing the distribution of oligodendrocytes (red), microglia (pink), and astrocytes (green) in sagittal brain sections of control, LPS-challenged, and CLP-induced mice. Blue represents DAPI staining. **B.** Mouse brain region map sourced from the Human Protein Atlas database (https://www.proteinatlas.org). **C–M.** Statistical analysis of the mean fluorescence intensity of each region of the brain in control, LPS-Challenged and CLP-induced mice. Statistical significance was calculated using the Mann–Whitney test, **p*<0.05, ***p*<0.01.

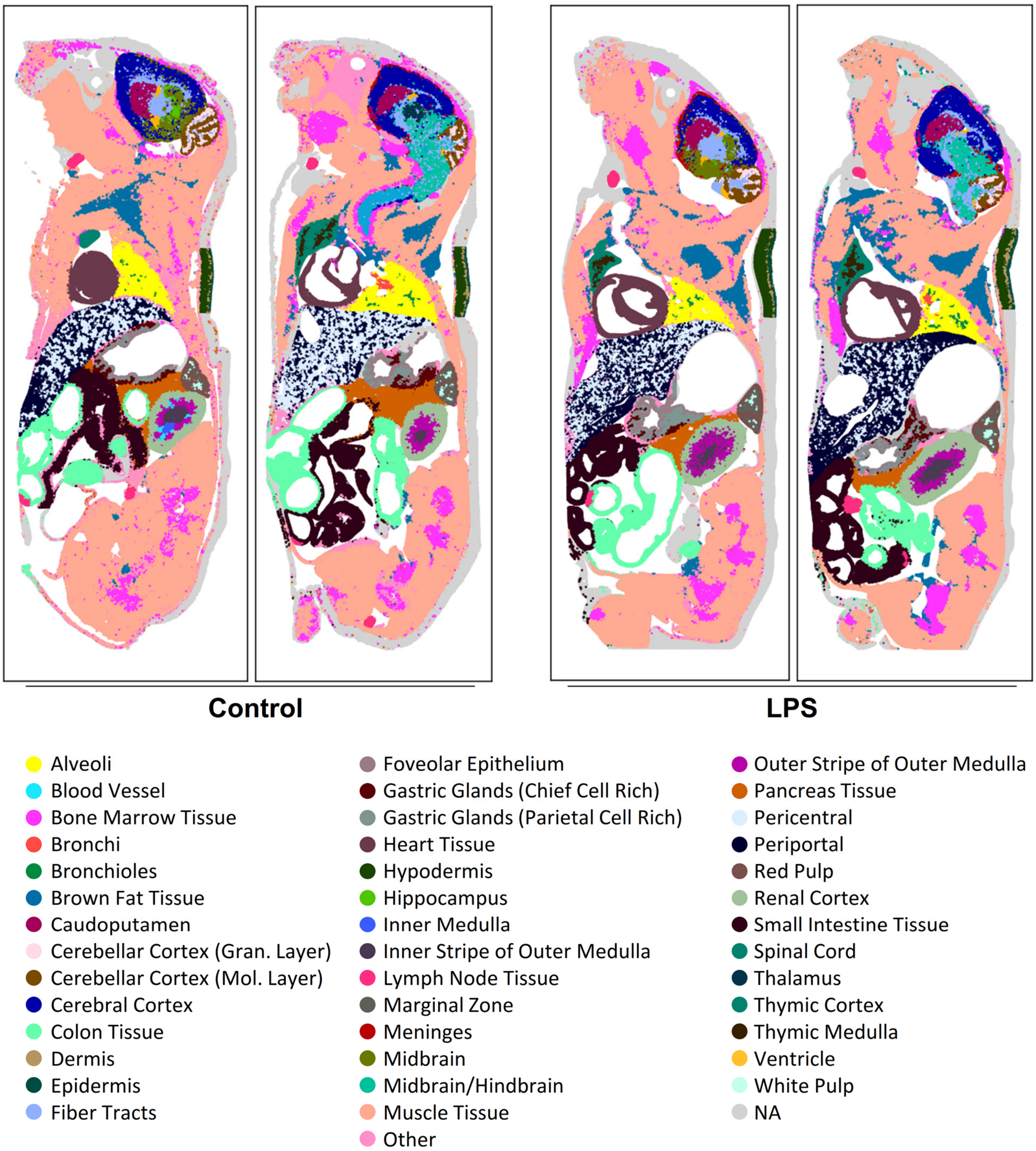

Supplementary Figure 6. Whole-mouse spatial transcriptomics annotation of organ overlaid on a hematoxylin and eosin (H&E) staining.

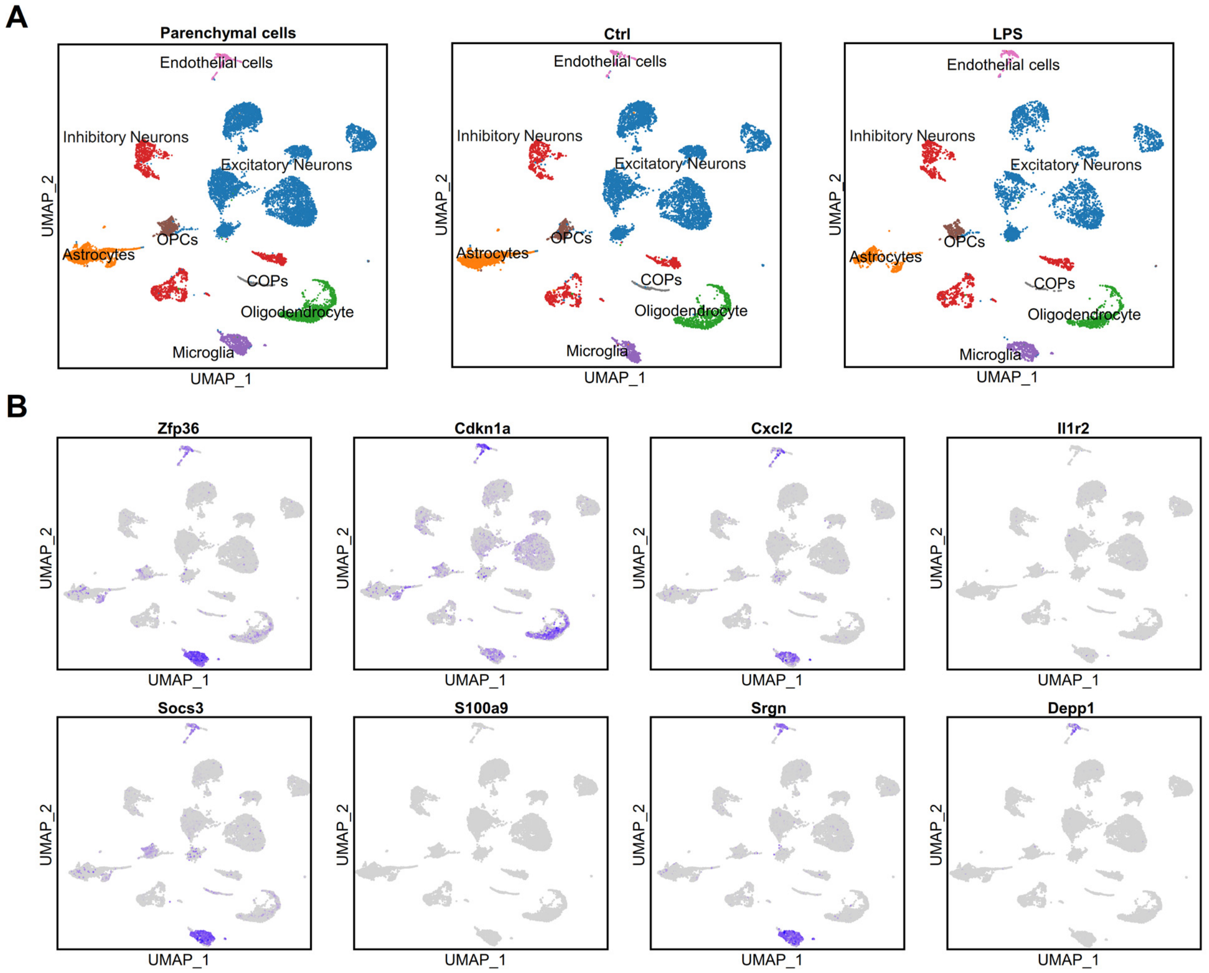

Supplementary Figure 7. Cell type validation and sepsis-related gene expression in the brain parenchymal cells of LPS-challenged mice brain. A. UMAP plots showing the distribution of various cell types in the motor cortex of wild-type (WT) mice under different conditions. All conditions together (left), LPS challenge group (middle), and PBS group (right) **B.** UMAP of eight represents sepsis-related gene expression colored by blue color intensity. UMAP, Uniform Manifold Approximation and Projection; LPS, lipopolysaccharide; PBS; phosphate-buffered saline.

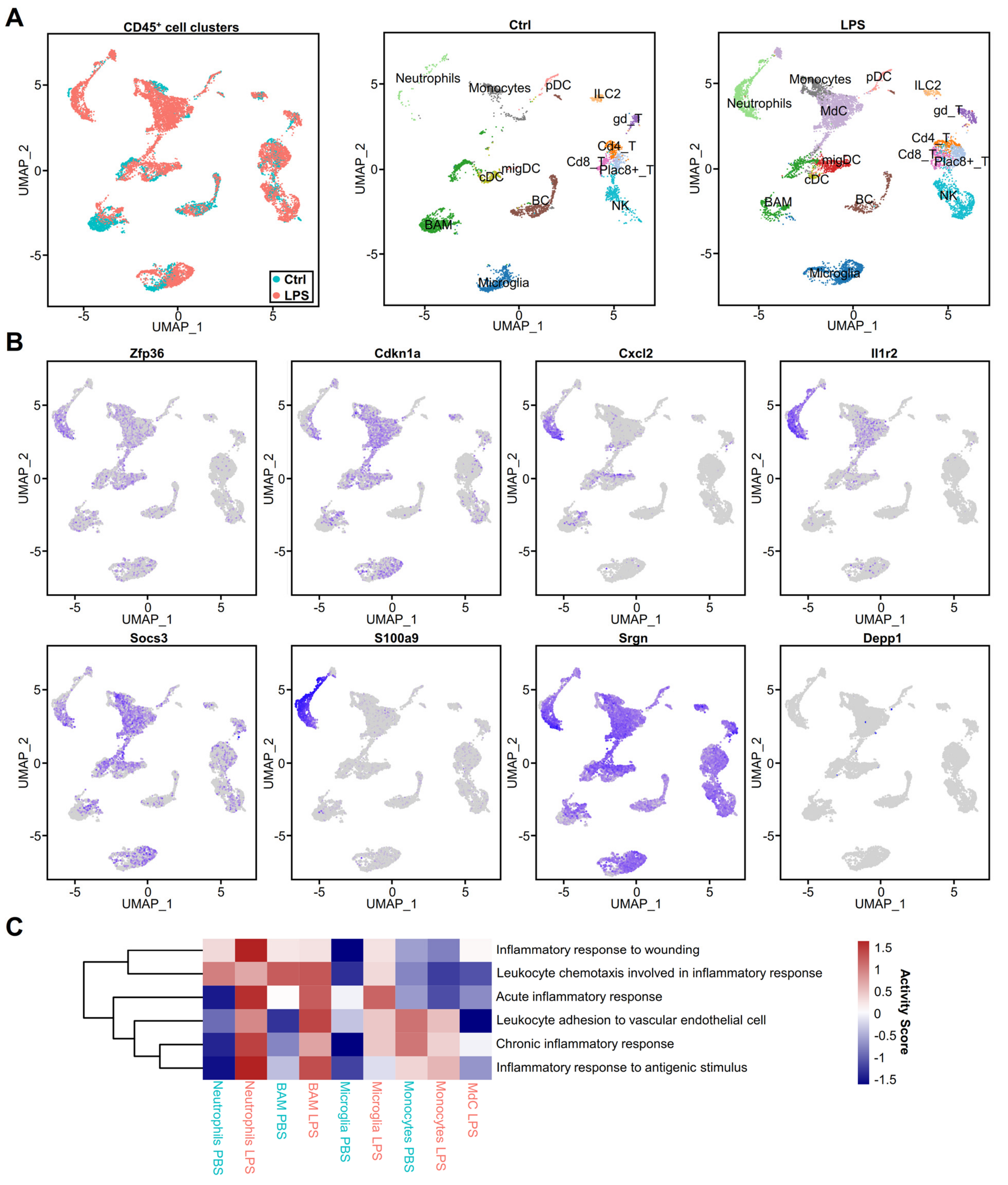

Supplementary Figure 8 Cell type validation and sepsis-related gene expression in the CD45+ cells of LPS challenged mice brain. A. UMAP plots show the difference in CD45+ cells isolated from whole brain (parenchyma and border regions) of mice post PBS or LPS challenge, color coded by both Ctrl and LPS group(left), only Ctrl group(middle) or only LPS group (right). **B.** UMAP of 8 represent sepsis-related genes expression colored by blue color intensity. **C.** Heatmap of GSVA enrichment scores showing differential pathway activities in Neutrophils, BAM, microglia, monocytes and MdC under LPS challenge.

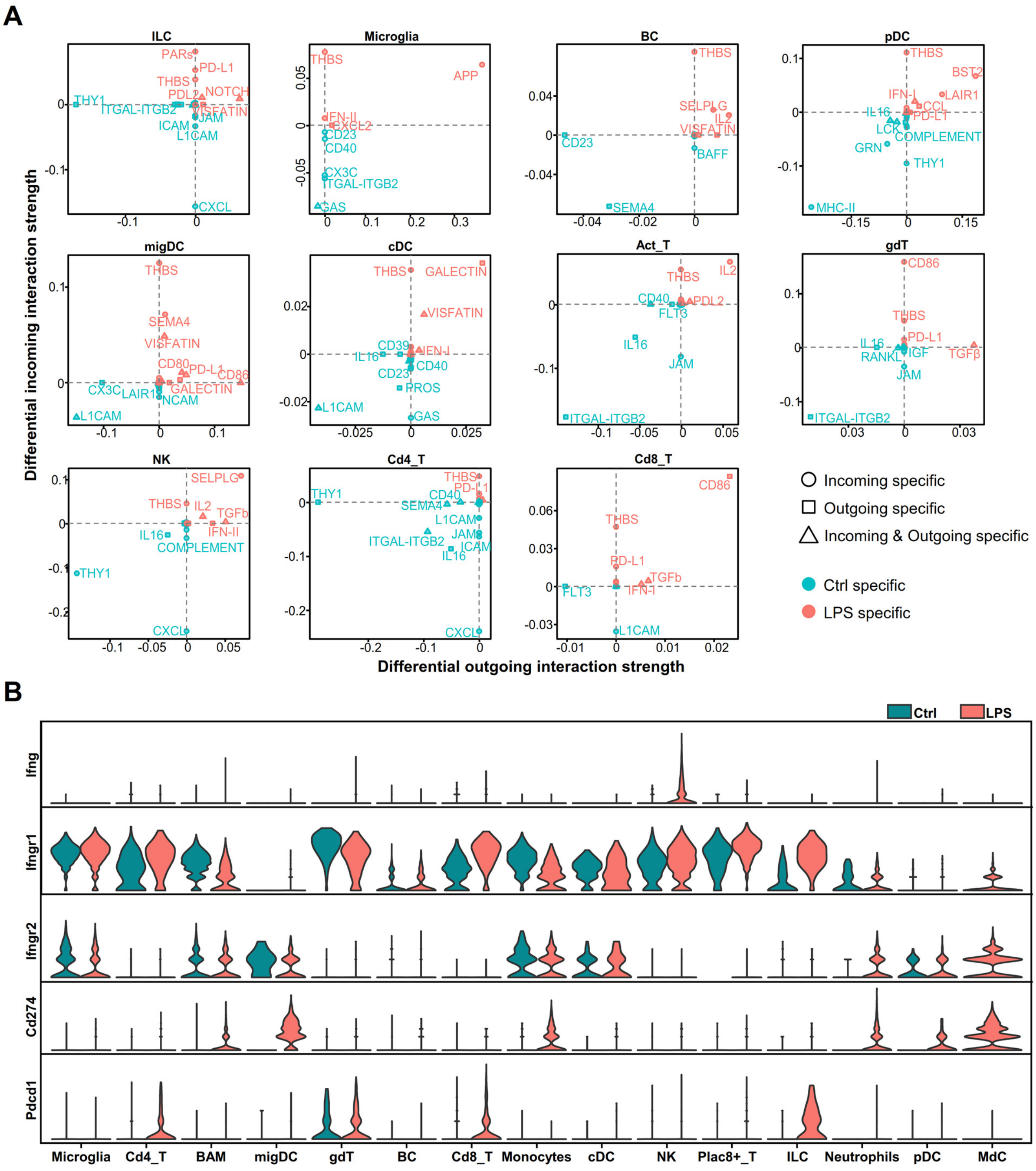

Supplementary Figure 9. The differential of outgoing (or incoming) signaling in CD45^{+} cells separated from the whole brain (parenchyma and border regions) of mice after PBS or LPS challenged. A. Scatter plot showing the outgoing (or incoming) signaling difference between representative CD45^{+} cells in the PBS (Ctrl) or LPS-challenged groups. **B.** Violin plot showing representative signaling pathway gene expression between PBS (Ctrl) or LPS challenged groups. LPS, lipopolysaccharide; PBS; phosphate-buffered saline.

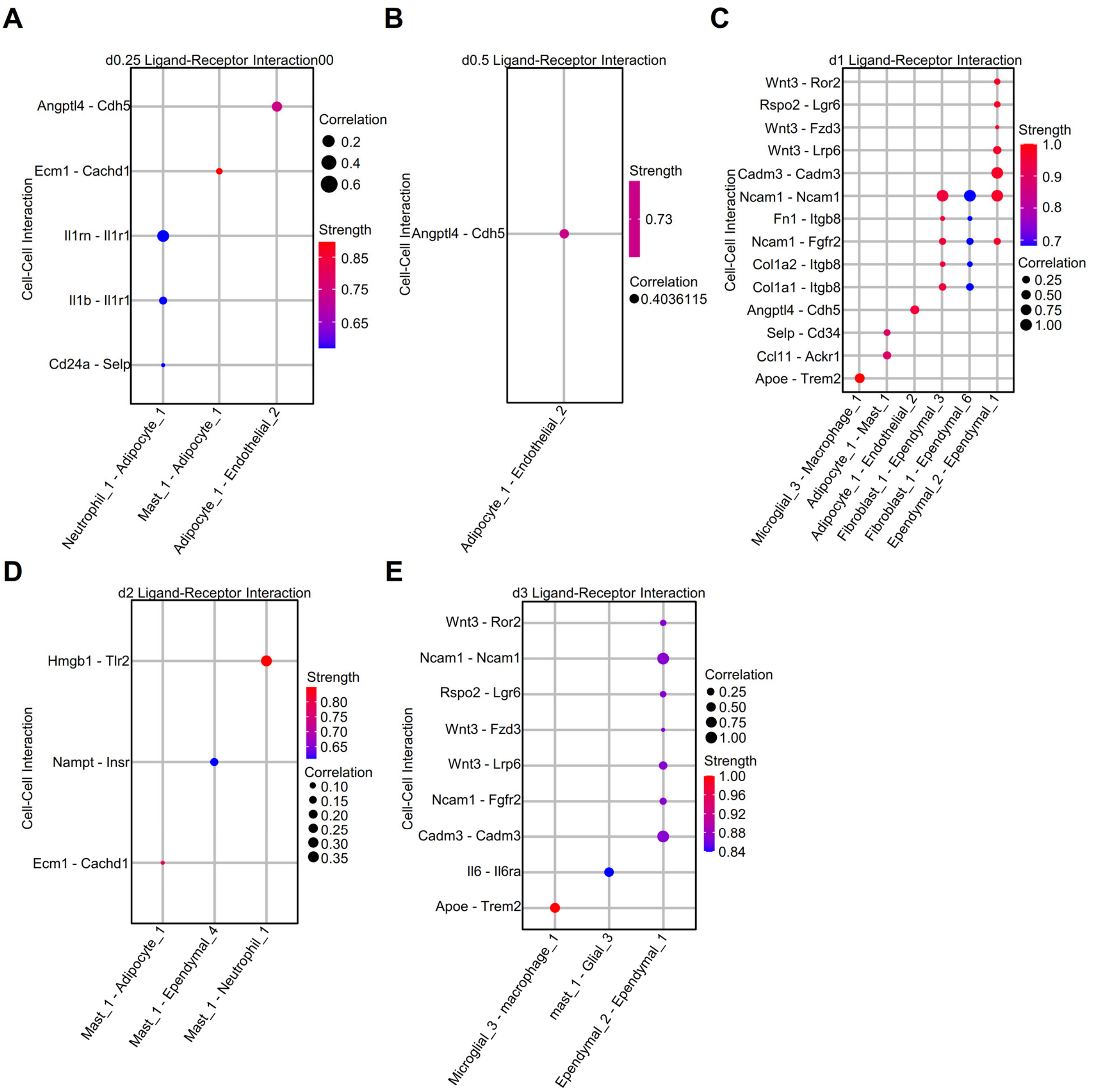

Supplementary Figure 10. Temporal dynamics of ligand-receptor interactions in mouse brain following LPS challenge. A–E. Dot plots showing the dynamics of ligand-receptor interactions in mouse brain at different time points following lipopolysaccharide (LPS) challenge. Each panel represents a specific time point post-LPS administration. The x-axis denotes interacting cell type pairs, while the y-axis shows ligand-receptor pairs mediating these interactions. Dot size corresponds to the correlation strength between ligand-receptor pairs, and color intensity indicates the interaction strength. **A** day 0.25; **B** day 0.5; **C** day 1; **D** day 2; **E** day 3 after LPS challenge.

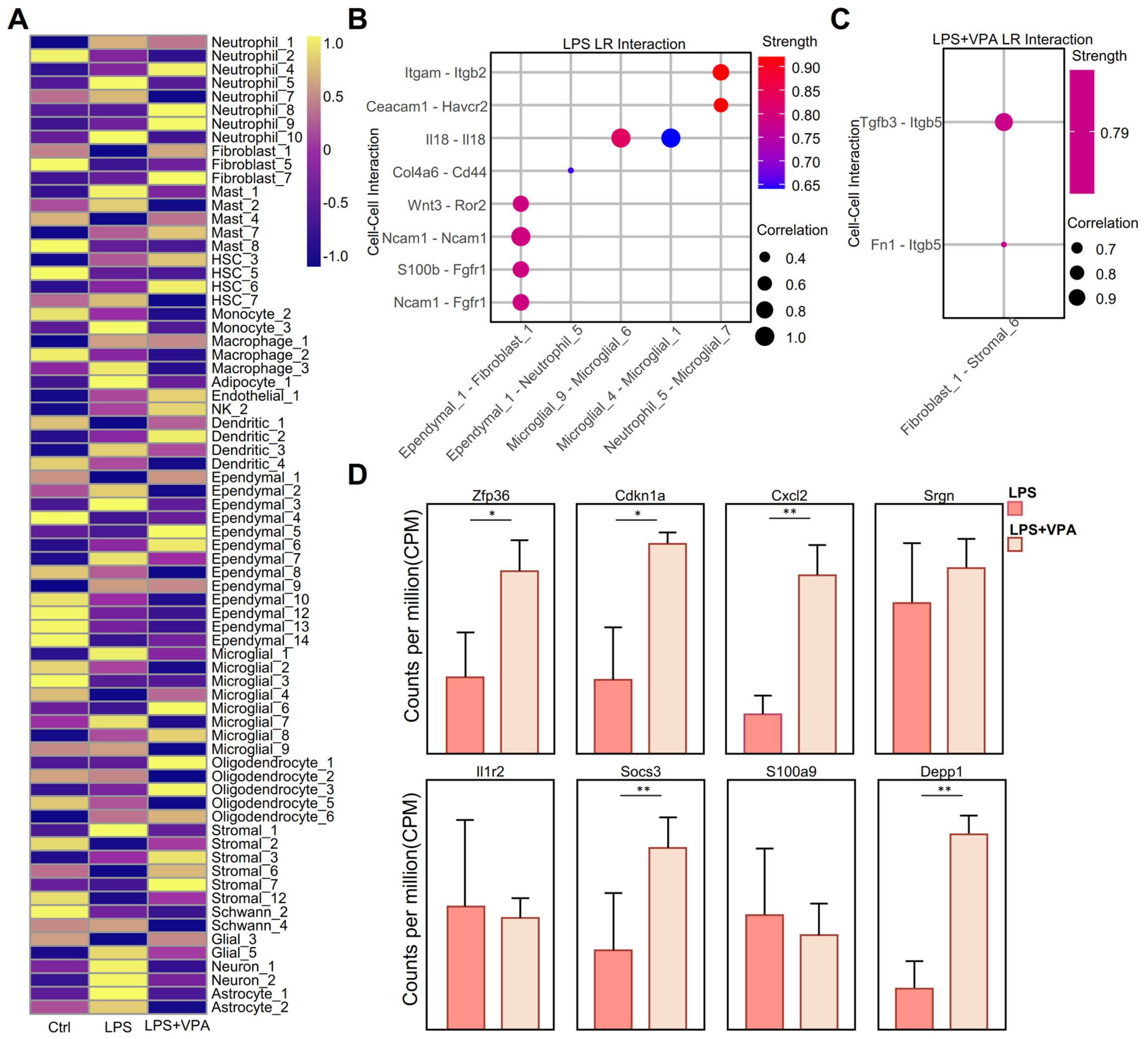

Supplementary Figure 11. Immune cell type deconvolution and inflammatory microenvironment network analysis in brain. A. Heatmap showing deconvolution analyses of cell subcluster between three groups. **B–C.** Dot plot shows ligand-receptor interactions in mouse brain following LPS challenge, without **(B)** or with **(C)** VPA treatment. **D.** CDEGs expression of LPS and LPS+VPA challenged RNA-seq data (*Limma*, *n=3*; *p < 0.05, **p < 0.01). LPS, lipopolysaccharide; CDEGs, conserved differentially expressed genes; VPA, valproic acid.

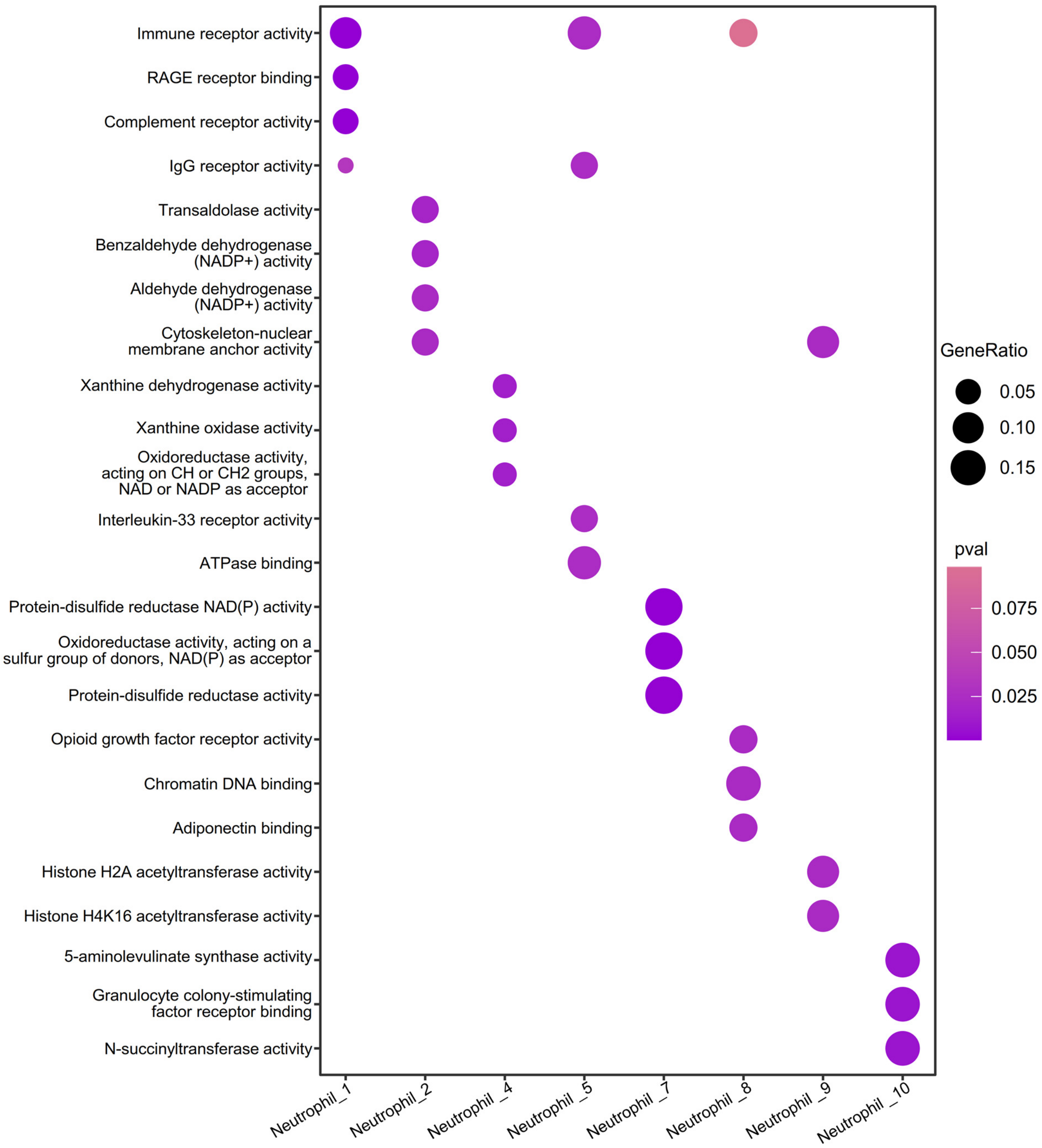

Supplementary Figure 12. Gene Ontology enrichment analyses of neutrophil subclusters specific genes estimated from deconvoluted analyses. Dot plot showing GO enrichment analyses of neutrophils subclusters specific genes.

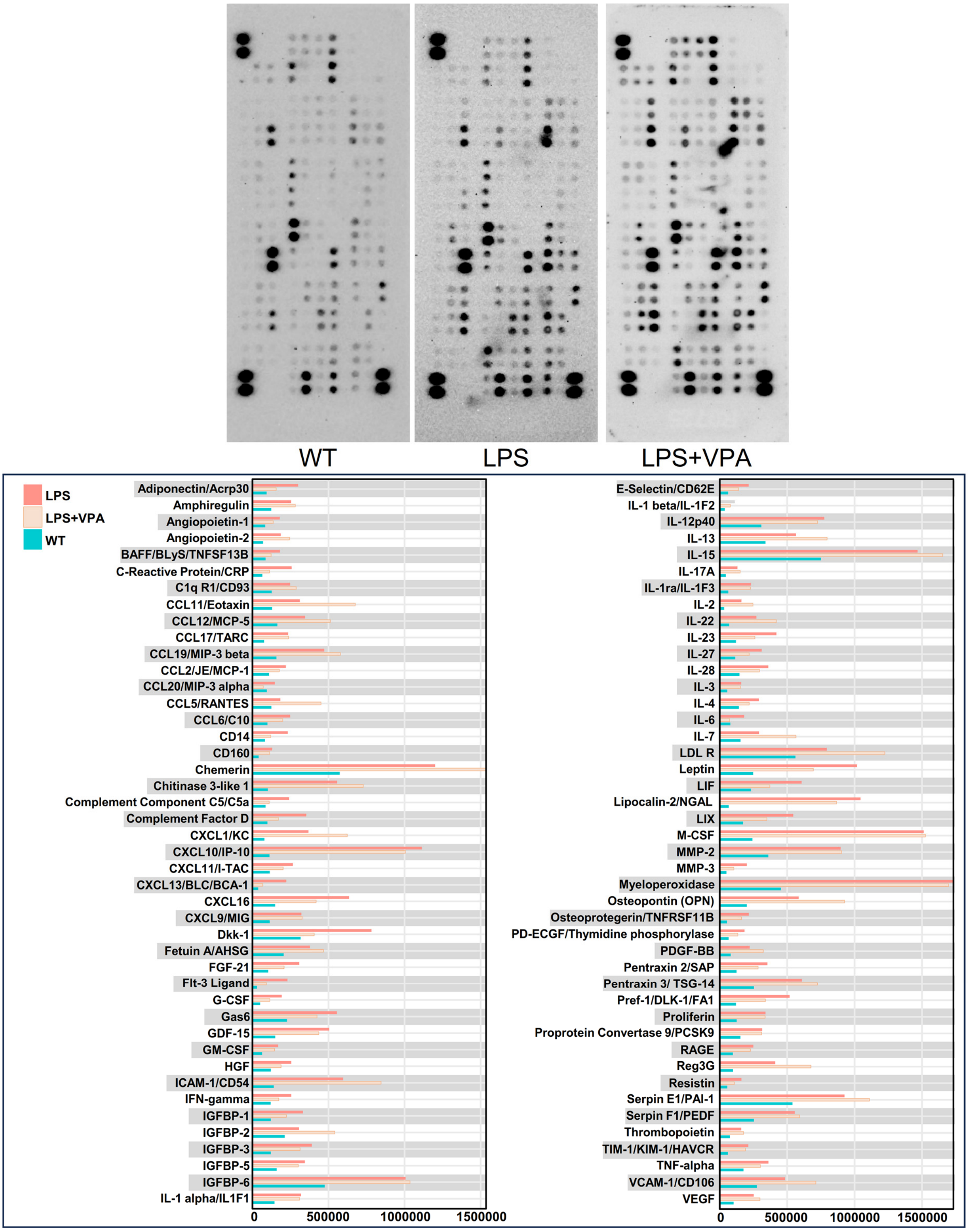

Supplementary Figure 13. Cytokine protein array analysis of WT, LPS, and LPS+VPA groups. The top panel shows representative images of the protein arrays for each group, with varying dot intensities indicating differential protein expression. Below, a bar graph quantifies the optical density measurements for numerous cytokines and related proteins across the three conditions. LPS, lipopolysaccharide; VPA, valproic acid; WT; wild-type.

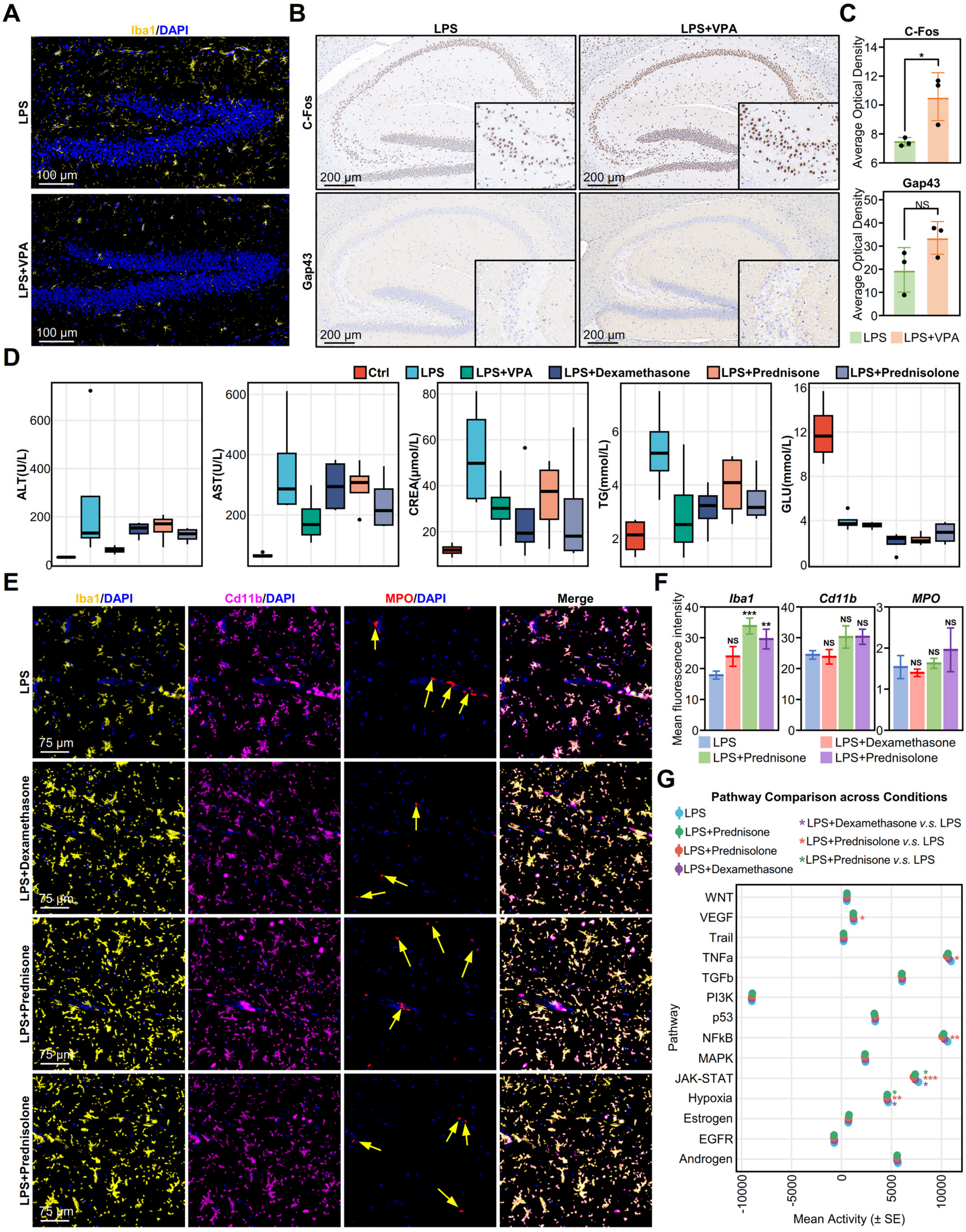

Supplementary Figure 14. Comparison of the effects of VPA and steroids in the treatment of septic brain. A. Immunofluorescence staining showing the distribution of microglial markers (yellow) in the hippocampus of mice 24 h after LPS or LPS+VPA injection. Blue represents DAPI

staining. **B.** Immunohistochemical staining of c-Fos and Gap43 in the hippocampus of mice 24 h after LPS or LPS+VPA injection. Insets show higher-magnification views of the indicated areas. **C.** Quantification of c-Fos and Gap43 optical density in the hippocampus. Statistical significance determined by unpaired *t*-test (n = 3 per group, *p < 0.05). **D.** Box plots showing serum biochemical parameters (ALT, AST, CREA, TG) across different challenge groups (control, LPS, LPS+VPA, LPS+Dexamethasone, LPS+Prednisone, LPS+Prednisolone; n = 4 per group). **E.** Immunofluorescence staining showing the distribution of microglial markers (Iba1: yellow, Cd11b: magenta) and the neutrophil marker (MPO: red) in sagittal brain sections of mice 24 h after LPS or LPS+ steroid (dexamethasone, prednisone, prednisolone) injection. Blue represents DAPI staining. Yellow arrows indicate neutrophil infiltration. **F.** Quantification of Iba1, Cd11b, and MPO immunofluorescence intensity under different treatment conditions. Statistical significance determined by ANOVA (n = 3 per group, *p < 0.05, **p < 0.01). **G.** RNA-seq pathway analysis comparing the molecular signatures across different treatment conditions. The dot plot shows the mean activity (±SE) of various signaling pathways including WNT, VEGF, TNF, p53, MAPK, and JAK-STAT pathways. Statistical significance determined by ANOVA (n = 5 per group, *p < 0.05, **p < 0.01, ***p < 0.001). VPA, valproic acid; LPS, lipopolysaccharide; DAPI, 4',6-diamidino-2-phenylindole; AST, aspartate transaminase; ALT, alanine transaminase; CREA, creatinine; TG, triglyceride; MPO, myeloperoxidase; Wnt, VEGF, vascular endothelial growth factor; TNF, tumor necrosis factor; MAPK, mitogen-activated protein kinase; JAK-STAT, Janus kinase/signal transducers and activators of transcription.

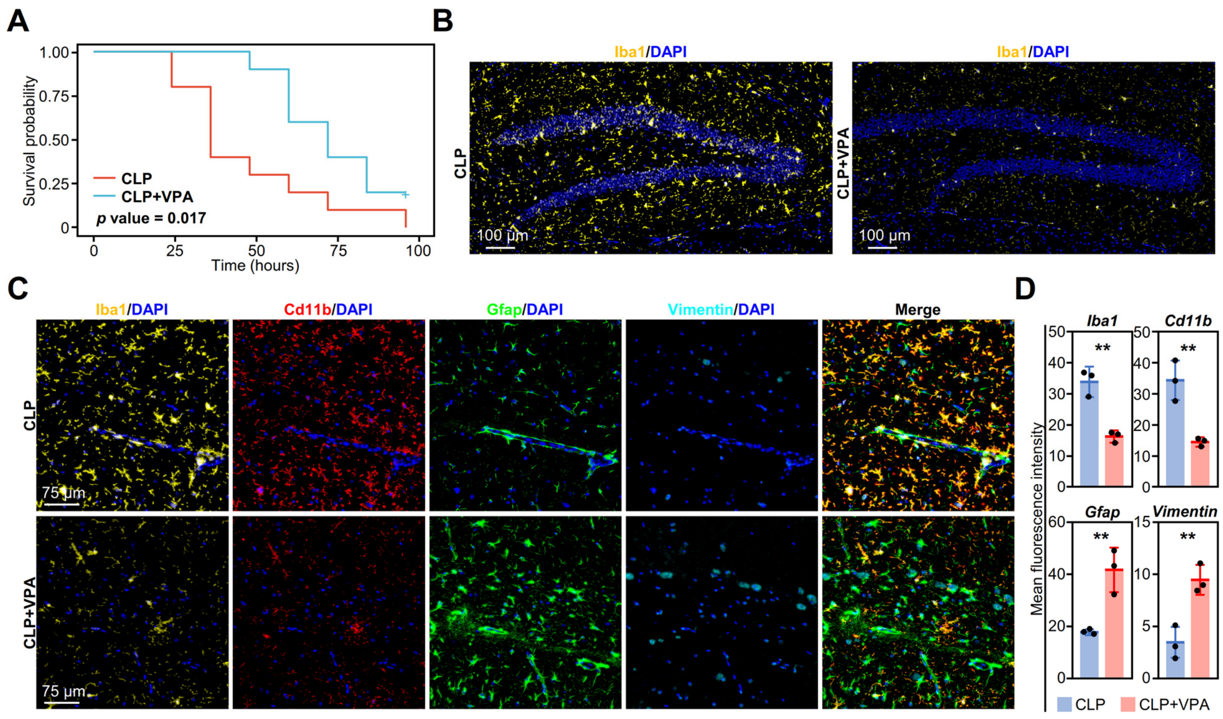

Supplementary Figure 15. Analysis of the therapeutic effect of VPA in CLP models. A. Kaplan–Meier survival curves of mice subjected to CLP with or without VPA treatment (n = 10 per group, p = 0.017). **B.** Representative immunofluorescence images showing microglial marker Iba1 (yellow) expression in the hippocampus of mice 24 h after CLP or CLP+VPA modeling. Nuclei were counterstained with DAPI (blue). **C.** Representative immunofluorescence images showing the distribution of microglial markers (Iba1: yellow, Cd11b: red) and astrocyte markers (Gfap: green, Vimentin: cyan) in the hippocampus of mice 24 h after CLP or CLP+VPA modeling. Arrows indicate Vimentin-positive cells. Nuclei were counterstained with DAPI (blue). **D.** Quantification of Iba1, Cd11b, Gfap, and Vimentin immunofluorescence intensity in CLP and CLP+VPA groups. Statistical significance determined by unpaired t-test (n = 3 per group, $^{**}p < 0.01$). VPA, valproic acid; CLP, cecal ligation and puncture; DAPI, 4',6-diamidino-2-phenylindole.

Supplementary Tables

Supplementary Table 1. qPCR primers for eight genes.

Gene name	Primer	Sequence
Depp1	Forward primer	5'-TCAGTGCTGGACAAGGTCACAG-3'
	Reverse primer	5'-CTGACGCAAAGAGAGCTGTCTC-3'
Srgn	Forward primer	5'-TGCGAACTGCATCGAGGAGAAG-3'
	Reverse primer	5'-CCCGAACCTGACCCATAGTCAT-3'
Cdkn1a	Forward primer	5'-TCGCTGTCTTGCACTCTGGTGT-3'
	Reverse primer	5'-CCAATCTGCGCTTGGAGTGATAG-3'
Zfp36	Forward primer	5'-TGTCGGACCTACTCAGAAAGCG-3'
	Reverse primer	5'-CTGGAGGTAGAACTTGTGGCAG-3'
Cxcl2	Forward primer	5'-CATCCAGAGCTTGAGTGTGACG-3'
	Reverse primer	5'-GGCTTCAGGGTCAAGGCAAACT-3'
Socs3	Forward primer	5'-GGACCAAGAACCTACGCATCCA-3'
	Reverse primer	5'-CACCAGCTTGAGTACACAGTCG-3'
Il1r2	Forward primer	5'-CAGTGCAGCAAGACTCTGGTAC-3'
	Reverse primer	5'-GCAAGTAGGAGACATGAGGCAG-3'
S100a9	Forward primer	5'-TGGTGGAAGCACAGTTGGCAAC-3'
	Reverse primer	5'-CAGCATCATACACTCCTCAAAGC-3'
18s	Forward primer	5'-AGTCCCTGCCCTTTGTACACA-3'
	Reverse primer	5'-CGATCCGAGGGCCTCACTA-3'

Supplementary Table 2. Sepsis-related genes of WGCNA analysis

No.	Gene name	No.	Gene name	No.	Gene name
1	*SLC11A1*	17	*CD93*	33	*S100A9*
2	*YBX3*	18	*PLSCR1*	34	*C10ORF10*
3	*NFKBIZ*	19	*GADD45B*	35	*GBP2*
4	*CEBPD*	20	*TEAD4*	36	*ADAMTS1*
5	*MT1L*	21	*LRRC32*	37	*ICAM1*
6	*ADAMTS9*	22	*C11ORF96*	38	*THBD*
7	*SRGN*	23	*CXCL2*	39	*IL1B*
8	*EMP1*	24	*CLEC2B*	40	*GPR4*
9	*CDKN1A*	25	*RP11-211G3.2*	41	*IFITM2*
10	*ZFP36*	26	*IL4R*	42	*C1R*
11	*PNP*	27	*RELL1*	43	*BTG2*
12	*SLCO4A1*	28	*SOCS3*	44	*SLA*
13	*BCL6*	29	*COL4A1*	45	*SECTM1*
14	*STC1*	30	*TNFRSF10D*	46	*DLL4*
15	*MPZL2*	31	*IRAK3*		
16	*FCGR2A*	32	*IL1R2*		

Supplementary Table 3. Regulator genes of genewalk analysis

No.	Gene name	No.	Gene name	No.	Gene name	No.	Gene name
1	*AMY2A*	15	*DTL*	29	*IL1B*	43	*PDLIM1*
2	*ANGPT2*	16	*FCGR2A*	30	*IL1R2*	44	*PLBD1*
3	*BAG3*	17	*FCGR3A*	31	*IL1RL1*	45	*PLP1*
4	*CACNA2D4*	18	*FCGR3B*	32	*IL4R*	46	*PNP*
5	*CD14*	19	*GADD45A*	33	*IL6*	47	*RAC2*
6	*CDK2*	20	*GADD45B*	34	*KRT19*	48	*S100A8*
7	*CDKN1A*	21	*GPR37*	35	*LCN2*	49	*S100A9*
8	*CFB*	22	*HCK*	36	*LDB3*	50	*SERPINH1*
9	*CISH*	23	*HSPB1*	37	*LY96*	51	*SIGLEC7*
10	*CMTM7*	24	*ICAM1*	38	*LYZ*	52	*SOCS3*
11	*CSF2RB*	25	*IFITM2*	39	*MGAM*	53	*TGM2*
12	*CSF3*	26	*IFITM3*	40	*MS4A4A*	54	*TNFRSF1A*
13	*CXCL2*	27	*IGFBP4*	41	*MUC5B*	55	*YBX3*
14	*CXCL3*	28	*IL18R1*	42	*OSMR*		

Note: Red represents sepsis-related genes.

Supplementary Table 4. Predicted phenotypes of *ZFP36*

Rank	Predicted mouse phenotypes (MGI*)	Predicted human phenotypes
1	Increased susceptibility to induced arthritis (MP:0003724)	Eczematoid dermatitis (HP:0000976)
2	Abnormal antigen presentation (MP:0001835)	Systemic lupus erythematosus (HP:0002725)
3	Peritoneal inflammation (MP:0003303)	Recurrent abscess formation (HP:0002722)
4	Decreased susceptibility to induced arthritis (MP:0003436)	Recurrent bacterial skin infections (HP:0005406)
5	Reproductive system inflammation (MP:0002254)	Hematochezia (HP:0002573)
6	Abnormal response to transplant (MP:0005671)	Myositis (HP:0100614)
7	Abnormal humoral immune (MP:0001800)	Elevated erythrocyte sedimentation rate (HP:0003565)
8	Abnormal immune tolerance (MP:0005000)	Glomerulonephritis (HP:0000099)
9	Immune system phenotype (MP:0005387)	Recurrent fungal infections (HP:0002841)
10	Abnormal immune system (MP:0001790)	Orchitis (HP:0100796)

*MGI: Mouse Genome Informatics database

Supplementary Table 5. Predicted phenotypes of *CDKN1A*

Rank	Predicted mouse phenotypes (MGI*)	Predicted human phenotypes
1	Abnormal cutaneous collagen (MP:0008438)	Abnormality of glycolipid metabolism (HP:0010969)
2	Peritoneal inflammation (MP:0003303)	Abnormality of liposaccharide metabolism (HP:0010968)
3	Abnormal hypodermis morphology (MP:0003705)	Abnormality of glycosphingolipid metabolism (HP:0004343)
4	Abnormal skin tensile (MP:0005275)	Premature rupture of membranes (HP:0001788)
5	Abnormal cell adhesion (MP:0003566)	Protrusio acetabuli (HP:0003179)
6	Decreased susceptibility to induced arthritis (MP:0003436)	Eczematoid dermatitis (HP:0000976)
7	Abnormal hypersensitivity reaction (MP:0002148)	Systemic lupus erythematosus (HP:0002725)
8	Gastrointestinal tract polyps (MP:0010352)	Loss of speech (HP:0002371)
9	Abnormal body composition (MP:0005451)	Vertebral compression fractures (HP:0002953)
10	Abnormal susceptibility to infection induced morbidity/mortality (MP:0009785)	Nuclear cataract (HP:0100018)

*MGI: Mouse Genome Informatics database

Supplementary Table 6. Predicted phenotypes of *CXCL2*

Rank	Predicted mouse phenotypes (MGI*)	Predicted human phenotypes
1	CNS inflammation (MP:0006082)	Recurrent abscess formation (HP:0002722)
2	Altered susceptibility to infection induced morbidity/mortality (MP:0009785)	Eczematoid dermatitis (HP:0000976)
3	Decreased susceptibility to induced arthritis (MP:0003436)	Stomatitis (HP:0010280)
4	Increased susceptibility to induced arthritis (MP:0003724)	Recurrent bacterial skin infections (HP:0005406)
5	Abnormal hypersensitivity reaction (MP:0002148)	Recurrent gram-negative bacterial infections (HP:0005420)
6	Peritoneal inflammation (MP:0003303)	Elevated erythrocyte sedimentation rate (HP:0003565)
7	Abnormal immune tolerance (MP:0005000)	Optic neuritis (HP:0100653)
8	Abnormal innate immunity (MP:0002419)	Retrobulbar optic neuritis (HP:0100654)
9	Abnormal antigen presentation (MP:0001835)	Orchitis (HP:0100796)
10	Abnormal hepatobiliary system (MP:0002139)	Mediastinal lymphadenopathy (HP:0100721)

*MGI: Mouse Genome Informatics database

Supplementary Table 7. Predicted phenotypes of *SOCS3*

Rank	Predicted mouse phenotypes (MGI*)	Predicted human phenotypes
1	Decreased susceptibility to induced arthritis (MP:0003436)	Cerebral aneurysm (HP:0004944)
2	Increased susceptibility to induced arthritis (MP:0003724)	Elevated erythrocyte sedimentation rate (HP:0003565)
3	Gastrointestinal ulcer (MP:0003300)	Orchitis (HP:0100796)
4	Myositis (MP:0004510)	Systemic lupus erythematosus (HP:0002725)
5	Reproductive system inflammation (MP:0002254)	Myositis (HP:0100614)
6	Abnormal susceptibility to infection induced morbidity/mortality (MP:0009785)	Loss of speech (HP:0002371)
7	Abnormal antigen presentation (MP:0001835)	Recurrent abscess formation (HP:0002722)
8	Skin inflammation (MP:0004947)	Hematochezia (HP:0002573)
9	Peritoneal inflammation (MP:0003303)	Cerebral aneurysm (HP:0004944)
10	Abnormal immune system (MP:0001790)	Disinhibition (HP:0000734)

*MGI: Mouse Genome Informatics database

Supplementary Table 8. Predicted phenotypes of *IL1R2*

Rank	Predicted mouse phenotypes (MGI*)	Predicted human phenotypes
1	Abnormal antigen presentation (MP:0001835)	Eczematoid dermatitis (HP:0000976)
2	Reproductive system inflammation (MP:0002254)	Recurrent abscess formation (HP:0002722)
3	Altered susceptibility to infection induced morbidity/mortality (MP:0009785)	Myositis (HP:0100614)
4	Decreased susceptibility to induced arthritis (MP:0003436)	Recurrent bacterial skin infections (HP:0005406)
5	Abnormal hypersensitivity reaction (MP:0002148)	Orchitis (HP:0100796)
6	Abnormal immune system (MP:0000685)	Retrobulbar optic neuritis (HP:0100654)
7	Peritoneal inflammation (MP:0003303)	Optic neuritis (HP:0100653)
8	Abnormal innate immunity (MP:0002419)	Recurrent gram-negative bacterial infections (HP:0005420)
9	Myositis (MP:0004510)	Spondylolisthesis (HP:0003302)
10	Abnormal response to transplant (MP:0005671)	Osteolytic defects of the hand bones (HP:0009699)

*MGI: Mouse Genome Informatics database

Supplementary Table 9. Predicted phenotypes of *S100A9*

Rank	Predicted mouse phenotypes (MGI*)	Predicted human phenotypes
1	Peritoneal inflammation (MP:0003303)	Recurrent gram-negative bacterial infections (HP:0005420)
2	Decreased susceptibility to induced arthritis (MP:0003436)	Recurrent bacterial skin infections (HP:0005406)
3	Abnormal peptide metabolism (MP:0003183)	Chronic obstructive pulmonary disease (HP:0006510)
4	Abnormal bone marrow (MP:0009278)	Obstructive lung disease (HP:0006536)
5	Abnormal innate immunity (MP:0002419)	Gingivitis (HP:0000230)
6	Abnormal susceptibility to infection induced morbidity/mortality (MP:0009785)	Mediastinal lymphadenopathy (HP:0100721)
7	Abnormal skeleton physiology (MP:0001533)	Abnormality of macrophages (HP:0004311)
8	Abnormal platelet physiology (MP:0005464)	Recurrent skin infections (HP:0001581)
9	Abnormal response to infection (MP:0005025)	Recurrent fungal infections (HP:0002841)
10	Eye inflammation (MP:0001851)	Optic neuritis (HP:0100653)

*MGI: Mouse Genome Informatics database

Supplementary Table 10. Predicted phenotypes of *SRGN*

Rank	Predicted mouse phenotypes (MGI*)	Predicted human phenotypes
1	Abnormal antigen presentation (MP:0001835)	Eczematoid dermatitis (HP:0000976)
2	Abnormal response to transplant (MP:0005671)	Myositis (HP:0100614)
3	Altered susceptibility to infection induced morbidity/mortality (MP:0009785)	Recurrent abscess formation (HP:0002722)
4	Abnormal immune system (MP:0000685)	Elevated erythrocyte sedimentation rate (HP:0003565)
5	Abnormal hypersensitivity reaction (MP:0002148)	Recurrent bacterial skin infections (HP:0005406)
6	Decreased susceptibility to induced arthritis (MP:0003436)	Thrombocytosis (HP:0001894)
7	Abnormal immune tolerance (MP:0005000)	Optic neuritis (HP:0100653)
8	Increased susceptibility to induced arthritis (MP:0003724)	Retrobulbar optic neuritis (HP:0100654)
9	Abnormal response to infection (MP:0005025)	Orchitis (HP:0100796)
10	Abnormal humoral immune (MP:0001800)	Petechiae (HP:0000967)

*MGI: Mouse Genome Informatics database

Supplementary Table 11. Predicted phenotypes of *DEPP1*

Rank	Predicted mouse phenotypes (MGI*)	Predicted human phenotypes
1	Abnormal chondrocyte physiology (MP:0009780)	Abnormality of aromatic amino acid family metabolism (HP:0004338)
2	Delayed dark adaptation (MP:0003011)	Complement deficiency (HP:0004431)
3	Abnormal lymphatic system (MP:0010368)	Systemic lupus erythematosus (HP:0002725)
4	Abnormal white adipose (MP:0005670)	Abnormality of complement system (HP:0005339)
5	Abnormal adipose tissue (MP:0000013)	Xanthomatosis (HP:0000991)
6	Abnormal hepatobiliary system (MP:0002139)	Hypoglycemic coma (HP:0001325)
7	Abnormal lipoprotein level (MP:0010329)	Joint hemorrhage (HP:0005261)
8	Abnormal blood-brain barrier (MP:0005167)	Increased CSF protein (HP:0002922)
9	Abnormal bile salt (MP:0005365)	Urinary glycosaminoglycan excretion (HP:0003541)
10	Abnormal cutaneous collagen (MP:0008438)	Mucopolysacchariduria (HP:0008155)

*MGI: Mouse Genome Informatics database

Supplementary Methods

RNA-seq data analysis

We conducted a comprehensive analysis of the sepsis-related changes in the brain using various analytical tools. To identify differences in gene function between septic and control human brains, we employed the Gene Set Variation Analysis (GSVA) [1], utilizing gene sets from the Molecular Signatures Database [2]. Significant differences were determined using Limma [3], with a *p*-value threshold of 0.05. Weighted Gene Correlation Network Analysis (WGCNA) [4] was used to identify modules and key genes related to clinical traits. We defined key genes as those with a high connectivity score (≥0.8). The module most closely associated with sepsis was further analyzed using ClueGO [5] to understand its biological role. We used BrainInABlender [6] to determine cell types in mouse and human brains and analyzed endocrine interactions between the brain and bone marrow using the QENIE [7] R script. For whole-body analysis of the lipopolysaccharide (LPS)-challenged mice, we processed Array-Seq data using Scanpy [8].

Seurat [9] is the primary tool for analyzing various types of single-cell RNA sequencing data, including single-nucleus (sn)RNA-seq, spatial RNA-seq, and smart-seq2. In our snRNA-seq analysis, we removed certain cells (ImOIG) that were significantly distant from the others in our dimensional reduction analysis **(Figure S1)**. We calculated scores for different gene sets, including those related to sepsis and specific cell types, and identified differentially expressed genes using Seurat's built-in functions. To gain a broader understanding of the gene function, we performed enrichment analyses using ClusterProfiler [10]. These included analyses of gene ontology (GO), biological pathways, and gene set enrichment. We used Limma to identify the differentially expressed genes (DEGs) in bulk RNA-seq samples from both humans and mice. GeneWalk [11] was used to identify genes with potential regulatory or multifunctional roles in sepsis. We focused on genes showing significant changes in expression ($|\log_2$ fold change$| \geq 0.6$, $p < 0.05$) in septic human brains compared to controls, considering results significant at a False

Discovery Rate (FDR) of 0.15.

Finally, we explored cell-to-cell communication in our single-nuclear RNA data using CellChat [12]. To gain insights into how systemic inflammation affects the mouse immune environment, we employed several specialized tools: Cytosig [13] to analyze cytokine activity, Progeny [14] to examine signaling pathways, and DeconvCellLink (https://github.com/JH-42/DeconvCellLink) to understand changes in cell subtypes, interactions between cells, and the interplay of receptors and ligands.

Immunofluorescence staining

The mice were euthanized by decapitation after cervical dislocation, and their brain tissues were immediately extracted and fixed in a 10% formalin solution. Paraffin sections were then microwaved for 23 min in ethylenediaminetetraacetic acid (EDTA) antigen retrieval solution (#G1206, Servicebio) and blocked with 3% bovine serum albumin at 25°C for 30 min. The tissue slides were then incubated with a specific primary antibody against Mbp (#D8X4Q, Cell Signaling Technology), Iba1 (#ab178846, Abcam) or Gfap (#sc-33673, Santa Cruz Biotechnology), Cd11b (#GB15058, Servicebio), Vimentin (#GB111308, Servicebio), Claudin-5 (#GB11290, Servicebio), vWF (#ab287962, Abcam), MPO (#15178, Cell Signaling Technology) overnight at 4 °C. After washing three times with phosphate-buffered saline (PBS), the slides were incubated with specific secondary antibodies: goat anti-rabbit IgG-horseradish peroxidase (HRP) (#GB23303, Servicebio), goat anti-rabbit IgG-fluorescein isothiocyanate (FITC) (#GB22303, Servicebio), or goat anti-mouse IgG-Cyanine5 (Cy5) (#GB27301, Servicebio) for 50 min. The slides were washed with PBS and stained with 4'-6-diamidino-2-phenylindole (DAPI) (#G1012, Servicebio), followed by incubation for 10 min in the dark. After counterstaining with DAPI, the slides were washed three times with PBS and covered with coverslips using an antifade mounting medium (#G1401, Servicebio).

Immunoblotting

Western blotting was performed in accordance with conventional procedures using specific primary antibodies against Mbp (#D8X4Q, Cell Signaling Technology), Iba1 (#ab178846, Abcam), Gfap (#sc-33673, Santa Cruz Biotechnology), and GAPDH (#cp. MB001, Bioworld), HRP-conjugated anti-rabbit IgG (#BS13278, Bioworld), and HRP-conjugated anti-mouse IgG (#BS12478, Bioworld). The gray values of the protein bands were quantified using ImageJ software (1.53e). Repeat measurements were performed to avoid bias induced by the operation, and the relative expression of proteins was determined by comparison with the internal reference protein, GAPDH.

Quantitative real-time PCR (qPCR)

Total RNA was extracted from the mouse forebrain using the TRIzol kit (#AAPR390-3, Pythonbio). Reverse transcription was performed using HiScript III RT SuperMix for qPCR (#R323-01, Vazyme). RT–qPCR was performed using Power Green qPCR Mix (#P2101a, GDSBio) under a specific cycling procedure, according to the manufacturer's instructions. Data were analyzed using the $2^{-\Delta\Delta Ct}$ method. The primer sequences are listed in Supplementary Table 1.

Hematoxylin and eosin (H&E) staining

Paraffin sections were dewaxed using Environmentally Friendly Dewaxing Transparent Liquids I and II for 20 min each, followed by dehydration in anhydrous ethanol and 75% ethanol for 5 min each, and rinsed with tap water. Frozen sections were fixed in tissue fixation solution for 15 min and rinsed with running water. The sections were pretreated with HD constant staining solution for 1 min, stained with hematoxylin for 5 min, differentiated with hematoxylin differentiation solution, and stained with hematoxylin bluing solution. After dehydration in ethanol and butanol, the sections were stained with eosin for 15 s, dehydrated in ethanol, cleared in xylene, and

mounted with neutral gum. The sections were observed under a light microscope.

Immunohistochemistry

Paraffin sections were dewaxed using environmentally friendly dewaxing solutions I, II, and III for 10 min each, followed by dehydration in anhydrous ethanol (I, II, and III for 5 min each) and rinsing in distilled water. Antigen retrieval was performed according to tissue-specific conditions, followed by washing in phosphate-buffered saline (PBS; pH 7.4) for three 5-min cycles. Endogenous peroxidase activity was blocked by incubating the sections in 3% hydrogen peroxide for 25 min, followed by washing in PBS. Subsequently, the sections were blocked with 3% bovine serum albumin (BSA) for 30 min. Primary antibodies against C-Fos (#GB11069, Servicebio) and Gap43 (#GB11095, Servicebio) were applied and incubated overnight at 4°C. After washing with PBS, the corresponding horseradish peroxidase (HRP)-labeled secondary antibody was applied and incubated at room temperature for 50 min. DAB was used for color development and the reaction was monitored under a microscope. The sections were then washed with tap water to stop the color development. Nuclei were counterstained with hematoxylin, differentiated, and blued with the appropriate solutions. Sections were dehydrated in graded alcohol, cleared in xylene, and mounted with an adhesive. Images were captured using a microscope.

Steroid treatment

To further investigate the differential effects of valproic acid (VPA) and steroids on brain immunomodulation during neuroinflammation, we administered three steroids—dexamethasone (HY-14648, MedChemExpress), prednisone (HY-B0214, MedChemExpress), and prednisolone (HY-17463, MedChemExpress)—to mice following LPS challenge. C57BL/6 mice were randomly assigned to either untreated or treatment groups. Untreated group: The mice were intraperitoneally injected with LPS (serotype O111:B4; Sigma-Aldrich) dissolved in Dulbecco's PBS (DPBS) at a dose of 10 mg/kg body weight. Treatment group: The mice were intraperitoneally

injected with 2 mg/kg dexamethasone, 20 mg/kg prednisone, or 20 mg/kg prednisolone 30 min after the initial LPS injection.

Supplementary References

1. Hanzelmann S, Castelo R, Guinney J. GSVA: gene set variation analysis for microarray and RNA-seq data. BMC Bioinformatics. 2013; 14: 7.
2. Liberzon A, Birger C, Thorvaldsdottir H, Ghandi M, Mesirov JP, Tamayo P. The Molecular Signatures Database (MSigDB) hallmark gene set collection. Cell Syst. 2015; 1: 417-425.
3. Ritchie ME, Phipson B, Wu D, Hu Y, Law CW, Shi W, et al. limma powers differential expression analyses for RNA-sequencing and microarray studies. Nucleic Acids Res. 2015; 43: e47.
4. Langfelder P, Horvath S. WGCNA: an R package for weighted correlation network analysis. BMC Bioinformatics. 2008; 9: 559.
5. Bindea G, Mlecnik B, Hackl H, Charoentong P, Tosolini M, Kirilovsky A, et al. ClueGO: a Cytoscape plug-in to decipher functionally grouped gene ontology and pathway annotation networks. Bioinformatics. 2009; 25: 1091-1093.
6. Hagenauer MH, Schulmann A, Li JZ, Vawter MP, Walsh DM, Thompson RC, et al. Inference of cell type content from human brain transcriptomic datasets illuminates the effects of age, manner of death, dissection, and psychiatric diagnosis. PLoS One. 2018; 13: e0200003.
7. Seldin MM, Koplev S, Rajbhandari P, Vergnes L, Rosenberg GM, Meng Y, et al. A Strategy for Discovery of Endocrine Interactions with Application to Whole-Body Metabolism. Cell Metab. 2018; 27: 1138-1155 e1136.
8. Wolf FA, Angerer P, Theis FJ. SCANPY: large-scale single-cell gene expression data analysis. Genome Biol. 2018; 19: 15.
9. Satija R, Farrell JA, Gennert D, Schier AF, Regev A. Spatial reconstruction of single-cell gene expression data. Nat Biotechnol. 2015; 33: 495-502.
10. Wu T, Hu E, Xu S, Chen M, Guo P, Dai Z, et al. clusterProfiler 4.0: A universal enrichment tool for interpreting omics data. Innovation (Camb). 2021; 2: 100141.
11. Ietswaart R, Gyori BM, Bachman JA, Sorger PK, Churchman LS. GeneWalk identifies relevant gene functions for a biological context using network representation learning. Genome Biol. 2021; 22: 55.
12. Jin S, Guerrero-Juarez CF, Zhang L, Chang I, Ramos R, Kuan CH, et al. Inference and analysis of cell-cell communication using CellChat. Nat Commun. 2021; 12: 1088.
13. Jiang P, Zhang Y, Ru B, Yang Y, Vu T, Paul R, et al. Systematic investigation of cytokine signaling activity at the tissue and single-cell levels. Nat Methods. 2021; 18: 1181-1191.
14. Schubert M, Klinger B, Klunemann M, Sieber A, Uhlitz F, Sauer S, et al. Perturbation-response genes reveal signaling footprints in cancer gene expression. Nat Commun. 2018; 9: 20.
